# A 3.5-minute-long reading-based fMRI localizer for the language network

**DOI:** 10.1162/IMAG.a.1246

**Published:** 2026-05-26

**Authors:** Greta Tuckute, Elizabeth Jiachen Lee, Aalok Sathe, Evelina Fedorenko

**Affiliations:** Department of Brain and Cognitive Sciences and McGovern Institute for Brain Research, Massachusetts Institute of Technology, Cambridge, MA, United States; Kempner Institute for the Study of Natural and Artificial Intelligence at Harvard University, Boston, MA, United States

**Keywords:** language comprehension, language network, speeded reading, rapid serial visual presentation, efficient functional localization, Multiple Demand network

## Abstract

The field of human cognitive neuroscience is increasingly acknowledging inter-individual differences in the precise locations of functional areas and the corresponding need for individual-level analyses in functional magnetic resonance imaging (fMRI) studies. One approach to identifying functional areas and networks within individual brains is based on robust and extensively validated ‘localizer’ paradigms—contrasts of conditions that aim to isolate some mental process of interest. Here, we present a new version of a localizer for the fronto-temporal language-selective network. This localizer is similar to a commonly used localizer based on the reading of sentences and nonword sequences but uses speeded presentation (200 ms per word/nonword). Based on a direct comparison between the standard version (450 ms per word/nonword) and the speeded version of the language localizer in 24 participants, we show that a single run of the speeded localizer (3.5 minutes) is highly effective at identifying the language-selective areas: indeed, it is more effective than the standard localizer given that it leads to an increased response to the critical (sentence) condition and a decreased response to the control (nonwords) condition. This localizer may therefore become the version of choice for identifying the language network in neurotypical adults or special populations (as long as they are proficient readers), especially when time is of essence.

## 1 Introduction

Neuroscientific studies of uniquely human abilities rely predominantly on non-invasive neuroimaging techniques such as functional magnetic resonance imaging (fMRI). A widespread methodological approach in fMRI studies of human brain function is to average individual activation maps for some contrast of interest in a template brain space and perform statistical analyses in each voxel across individuals to derive a group-level whole-brain statistical map. However, functional regions vary in their precise locations across individuals (Fischl et al., 2008; Frost & Goebel, 2012; Somers et al., 2021; Tahmasebi et al., 2012; Vázquez-Rodríguez et al., 2019). Correspondingly, reliance on these group-averaging approaches can lead to low sensitivity and functional resolution (Brett et al., 2002; Nieto-Castañón & Fedorenko, 2012; Saxe et al., 2006). Inter-individual variability is particularly problematic when functional regions of interest lie in proximity to functionally distinct regions, as is the case with both frontal and temporal language regions (e.g., Braga et al., 2020; Deen et al., 2015; Du et al., 2024; Fedorenko et al., 2012; Tahmasebi et al., 2012; Tomaiuolo et al., 1999; see Fedorenko & Blank, 2020, for discussion of this issue for ‘Broca’s area’).

One increasingly popular solution that circumvents inter-individual variability in the precise locations of functional regions is the use of functional ‘localizers’ (Gratton & Braga, 2021; Nieto-Castañón & Fedorenko, 2012; Saxe et al., 2006). In this approach, a brain region or network that supports a mental process of interest is identified with a functional contrast in each individual brain and subsequently, the region’s/network’s responses to some critical condition(s) of interest are examined. Historically, localizer development has been guided by dissociations in patients with brain damage or in behavioral investigations. For example, a double dissociation between face and object recognition (McMullen et al., 2000; Moscovitch et al., 1997) and clearly distinct behavioral signatures of processing faces vs. non-face objects (McKone et al., 2001; McNeil & Warrington, 1993) have motivated the *faces > objects* localizer contrast (Kanwisher et al., 1997). However, in principle, any contrast that (a) isolates some aspect of perception, motor control, or cognition, (b) elicits robust and replicable activation at the individual-participant level, and (c) identifies spatially consistent areas across individuals can be adopted as a localizer. The critical feature of localizers is that they are used consistently across studies and labs (and in some cases, species; Russ et al., 2021). This consistent use affords greater confidence that the ‘same’ functional region or set of regions is being studied, compared to relying on anatomical landmarks alone, and thus facilitates the accumulation of scientific knowledge.

The functional localization approach has been successful across many domains of perception and cognition including high-level visual and auditory processing, social cognition, and language (Baker et al., 2007; Belin et al., 2002; Downing et al., 2001; Epstein & Kanwisher, 1998; Fedorenko et al., 2010, 2013; Fischer et al., 2016; Isik et al., 2017; Kanwisher et al., 1997; Overath et al., 2015; Saxe & Kanwisher, 2003). In the domain of language, Fedorenko et al. (2010) developed a localizer that relies on a contrast between language processing and the processing of a perceptually similar condition that lacks linguistic structure or meaning (e.g., reading or listening to sentences vs. nonword lists, or listening to sentences vs. backwards speech or acoustically degraded sentences; Bedny et al., 2011; Lipkin et al., 2022; Malik-Moraleda, Ayyash et al., 2022; Scott et al., 2017). Such contrasts target brain areas that support computations related to accessing words and combining them into complex linguistic structures and meanings. These ‘language localizers’ robustly identify the left-lateralized fronto-temporal language network, which has long been implicated in language processing based on investigations of patients with aphasia (e.g., Bates et al., 2003; Fridriksson et al., 2018; Goodglass, 1993; Luria, 1970; Wilson et al., 2023) and group-averaging neuroimaging investigations of language processing (e.g., Binder et al., 1997; Friederici, 2012; Price, 2010). Importantly, language localizers are highly generalizable, eliciting similar activations across presentation modalities, materials, and tasks (see Fedorenko et al., 2024). Moreover, the brain regions that this localizer identifies closely correspond to those that emerge from the bottom-up clustering of voxel time-courses obtained during rest (Braga et al., 2020) or while performing tasks (Du et al., 2025; Shain & Fedorenko, 2025). This correspondence highlights that the language network is a ‘natural kind’ in the brain: an ontologically meaningful grouping of a set of brain regions that show highly synchronized activity over time. Neuroimaging studies of language that rely on the functional localization approach have produced a number of robust and replicable findings both about (i) the relationship between language and other perceptual and cognitive processes (e.g., Amalric & Dehaene, 2019; Chen et al., 2023; Deen et al., 2015; Fedorenko et al., 2011; Ivanova et al., 2020; Jouravlev et al., 2019; Shain et al., 2023), and (ii) the internal organization of the language system and its computations (e.g., Blank et al., 2016; Fedorenko et al., 2020; Shain et al., 2022, 2024).

One practical concern that researchers often express about the use of localizers is that they take time. Time is often a precious commodity in neuroimaging research, either because the critical task is already long and/or because the population of interest may have low tolerance for the scanner environment. However, given the advantages that localizers provide—including greater sensitivity, greater functional resolution, more accurate effect size estimation, higher interpretability of the responses in the critical tasks, and the ability to meaningfully accumulate knowledge across studies, labs, and species—many researchers continue to adopt this approach. One recent effort in the field has therefore been to try to optimize localizers so that they can be as short as possible while still yielding robust individual-level responses (e.g., Dodell-Feder et al., 2011; Lee et al., 2024; Marvi, Hutchinson et al., 2025).

In this study, we develop a shorter version of a widely used reading-based language localizer (Fedorenko et al., 2010). We leverage the fact that humans can read at fast rates, especially when the need for eye movements is minimized by presenting words one at a time in the center of the screen in a rapid serial visual presentation (RSVP) paradigm (e.g., Forster, 1970; Mollica & Piantadosi, 2017; Potter, 2012; Potter et al., 1980, 1986). In these studies, participants can process linguistic information even when each word is presented for as little as ~80–200 ms, as evidenced by accurate recall of the stimuli and high accuracy in answering comprehension questions about the content. Moreover, a few studies (Benjamin & Gaab, 2012; Christodoulou et al., 2014; Vagharchakian et al., 2012) have found that speeded reading, similar to reading at slower speeds, activates the language areas, but these studies have used a group-averaging approach, leaving open the question of whether speeded reading elicits sufficiently robust responses in individual participants. This is the question our study aims to address. Although this question is primarily methodological in nature, our study’s design allows us to additionally ask a theoretically interesting question about whether the increased processing difficulty due to speeded presentation affects neural responses in the language-selective network, or instead (or in addition) in the domain-general Multiple Demand network, which is sensitive to cognitive effort across diverse paradigms (e.g., Assem, Glasser, et al., 2020; Duncan, 2010; Duncan et al., 2012, 2020; Fedorenko et al., 2013).

## 2 Methods

### 2.1 Brief overview

Twenty-four adults each completed two versions of a language localizer task. In both versions, participants read sentences and lists of unconnected pronounceable nonwords presented on the screen one word/nonword at a time. The two versions differed in the presentation speed of each word/nonword. One localizer version was an extensively validated language localizer task (Fedorenko et al., 2010; Mahowald & Fedorenko, 2016; see Lipkin et al., 2022 for data from >600 participants on this version) where each word/nonword is presented for 450 ms (‘standard language localizer’). The other version was a new, speeded version of the task where each word/nonword was presented for 200 ms (‘speeded language localizer’). Twenty-two of the 24 participants completed the two versions of the language localizer in the same scanning session; the remaining two—in separate sessions (1 and 463 days apart). For all participants, the speeded version was run after the standard version. Each scanning session lasted between 1 and 2 hours and included a variety of additional tasks for unrelated studies. The materials, scripts, and screen recordings for the two language localizer versions are available at https://www.evlab.mit.edu/resources-all/download-localizer-tasks (standard version) and https://github.com/el849/speeded_language_localizer/ (speeded version).

### 2.2 Participants

Twenty-four neurotypical adults (12 female, 12 male), aged 18 to 60 (mean: 28.04; std: 9.25), participated for payment between June 2021 and December 2022. All participants were native speakers of English, had normal or corrected-to-normal vision, and no history of neurological, developmental, or language impairments. Twenty-two participants (~92%) were right-handed, as determined by the Edinburgh handedness inventory (Oldfield, 1971), 2 participants (~8%) were left-handed. All participants gave informed written consent in accordance with the requirements of the MIT’s Committee on the Use of Humans as Experimental Subjects (COUHES).

### 2.3 fMRI tasks

#### 2.3.1 Language network localizer tasks

##### 2.3.1.1 Standard language localizer task

A reading task contrasted *sentences* (e.g., THE SPEECH THAT THE POLITICIAN PREPARED WAS TOO LONG FOR THE MEETING) and lists of unconnected, pronounceable *nonwords* (e.g., LAS TUPING CUSARISTS FICK PRELL PRONT CRE POME VILLPA OLP WORNETIST CHO) in a standard blocked design with a counterbalanced condition order across runs, as introduced in Fedorenko et al. (2010). Each stimulus consisted of 12 words/nonwords. Stimuli were presented in the center of the screen, one word/nonword at a time, at the rate of 450 ms per word/nonword. Each stimulus was preceded by a 100 ms blank screen and followed by a 400 ms screen showing a picture of a finger pressing a button, and a blank screen for another 100 ms, for a total trial duration of 6 seconds. Participants were instructed to read attentively (silently, to themselves) and to press a button on the button box whenever they saw the picture of a finger pressing a button on the screen. The button-pressing task was included to help participants remain alert. Experimental blocks lasted 18 seconds (with 3 trials per block) and fixation blocks lasted 14 seconds. Each run (consisting of 16 experimental blocks and 5 fixation blocks) lasted 358 seconds (5 minutes 58 seconds). Participants completed 2 runs.

##### 2.3.1.2 Speeded language localizer task

The speeded version of the language localizer was identical to the standard version except that each word/nonword was presented for 200 ms instead of 450 ms (i.e., ~56% shorter). Each stimulus was preceded by a 100 ms blank screen and followed by a 400 ms screen showing a picture of a finger pressing a button, and a blank screen for another 100 ms, for a total trial duration of 3 seconds. The instructions to the participants were the same as in the standard version although they were warned that the presentation would be somewhat fast, and they were told not to worry if they missed some button presses. Experimental blocks lasted 9 seconds (with 3 trials per block) and fixation blocks lasted 14 seconds. Each run (consisting of 16 experimental blocks and 5 fixation blocks) lasted 214 seconds (3 minutes 34 seconds). Participants completed 2 runs.

#### 2.3.2 Language network localizer experimental materials

##### 2.3.2.1 Standard language localizer materials

The materials consisted of five sets, each set comprising 48 sentences and 48 nonword sequences, for a total of 240 sentences and 240 nonword sequences. The sentences were drawn from the Brown corpus (Bird & Loper, 2004; Francis & Kucera, 1964) and were selected to include a variety of syntactic constructions and topics. The nonwords were created using the ‘Wuggy’ software (https://github.com/WuggyCode/wuggy; the default parameters were used) so as to respect the phonotactic constraints of English. In cases where Wuggy was unable to generate a nonword candidate, we relied on one of the following strategies: (i) broke down the word into composite words (for compound words) or morphemes, matched each composite word/morpheme to a nonword, and then reassembled those; (ii) used one of the nonwords created for another word; or (iii) created an English-sounding nonword ourselves. Any given participant saw one set of materials.

##### 2.3.2.2 Speeded language localizer materials

The first 11 participants were presented with the materials from the standard version (ensuring that a different set was used). Approximately halfway through data collection, we created a new set of materials for the speeded language localizer in order to: (i) generalize the findings to a new set of materials, and (ii) avoid potential material overlaps between the standard and speeded localizer materials in future experiments. Hence, for the remaining 13 participants, we created five new sets each consisting of 48 sentences and 48 nonword sequences, for a total of 240 new sentences and 240 new nonword sequences. The sentences were again selected from the Brown corpus (Bird & Loper, 2004). In particular, we sampled 1,000 12 word-long sentences and then selected a set of 240 sentences that were not already included in the original set of materials, were syntactically and semantically diverse, and did not contain offensive/inappropriate content. The nonword strings were created as in the standard version.

#### 2.3.3 Multiple Demand network localizer task

In addition to the language tasks, we included a non-linguistic demanding task: a spatial working memory task. The goal was two-fold. First, including a non-linguistic task allowed us to evaluate the *selectivity* of the language fROIs—defined by two versions of the localizer—for language processing (Fedorenko et al., 2011, 2024). And second, this task allowed us to examine brain responses to the conditions of the language localizer tasks in *another set of functional areas*: areas that comprise the domain-general Multiple Demand (MD) network (Duncan, 2010; Duncan et al., 2012; Fedorenko et al., 2013). This network supports executive functions like working memory and cognitive control. The spatial WM task has been previously established to robustly identify these areas at the individual-participant level (e.g., Assem, Blank, et al., 2020; Blank et al., 2014; Malik-Moraleda, Ayyash et al., 2022; Mineroff, Blank et al., 2018; Shashidhara et al., 2020). Although the areas of the MD network have been shown to not support any ‘core’ linguistic computations—like those related to lexical access, syntactic structure building, or semantic composition (e.g., Blank & Fedorenko, 2017; Diachek, Blank, Siegelman et al., 2020; Quillen et al., 2021; Shain et al., 2020, 2022)—their engagement has been reported for some cases of effortful perception and comprehension (e.g., Liu et al., 2022; MacGregor et al., 2022; Mattys & Wiget, 2011; see Discussion). We therefore wanted to evaluate the MD areas’ responses to speeded comprehension, to see whether this kind of processing difficulty draws on domain-general resources.

The spatial working memory task contrasted a *hard* condition with an *easy* condition in a standard blocked design with a counterbalanced condition order across runs (e.g., Blank et al., 2014; Fedorenko et al., 2011, 2013). On each trial (duration = 8 seconds), participants saw a fixation cross for 500 ms, followed by a 3 x 4 grid within which randomly generated locations were sequentially flashed (1 second per flash) two at a time for a total of eight locations (*hard* condition) or one at a time for a total of four locations (*easy* condition). Then, participants indicated their memory for these locations in a two-alternative forced-choice paradigm via a button press (the choices were presented for 1,000 ms, and participants had up to 3 seconds to respond). Feedback, in the form of a green checkmark (correct responses) or a red cross (incorrect responses), was provided for 250 ms, with fixation presented for the remainder of the trial. Experimental blocks lasted 32 seconds (with 4 trials per block) and fixation blocks lasted 16 seconds. Each run (consisting of 12 experimental blocks and 4 fixation blocks) lasted 448 seconds (7 minutes 28 seconds). Participants completed 2 runs.

Twenty-three of the 24 participants completed the MD localizer in the same scanning session as the standard language localizer; the remaining participant—in a separate session (98 days apart).

### 2.4 fMRI data acquisition, preprocessing and first-level analysis

#### 2.4.1 fMRI data acquisition

Structural and functional data were collected on the whole-body, 3 Tesla, Siemens Trio scanner 32-channel head coil, at the Athinoula A. Martinos Imaging Center at the McGovern Institute for Brain Research at MIT. T1-weighted, Magnetization Prepared RApid Gradient Echo (MP-RAGE) structural images were collected in 176 sagittal slices with 1 mm isotropic voxels (TR = 2,530 ms, TE = 3.48 ms, TI = 1,100 ms, flip = 8 degrees). Functional, blood oxygenation level dependent (BOLD) data were acquired using one of three similar sequences (denoted as sequence A, B, C). The data from the majority of participants (22 out of 24) were acquired using sequence A which we describe in this paragraph. See specifications of sequences B and C in Supplementary Table S1 (importantly, scanning sequence is kept constant in all comparisons between the standard and the speeded versions of the localizer besides in a single participant). Sequence A was an SMS EPI sequence (with a 90 degree flip angle and using a slice acceleration factor of 2), with the following acquisition parameters: fifty-two 2 mm thick near-axial slices acquired in the interleaved order (with 10% distance factor), 2 mm × 2 mm in-plane resolution, FoV in the phase encoding (A ≫ P) direction 208 mm and matrix size 104 × 104, TR = 2,000 ms and TE = 30 ms, and partial Fourier of 7/8. The first 10 seconds of each run were excluded to allow for steady state magnetization.

#### 2.4.2 fMRI preprocessing

fMRI data were analyzed using SPM12 (release 7487), CONN EvLab module (release 19b; https://web.conn-toolbox.org/resources/conn-extensions/evlab), and custom MATLAB scripts. Each participant’s functional and structural data were converted from DICOM to NIfTI format. All functional scans were coregistered and resampled using B-spline interpolation to the first scan of the first session (Friston et al., 1995). Potential outlier scans were identified from the resulting subject-motion estimates as well as from BOLD signal indicators using default thresholds in CONN preprocessing pipeline (5 standard deviations above the mean in global BOLD signal change, or framewise displacement values above 0.9 mm; (Nieto-Castanon, 2020). Functional and structural data were independently normalized into a common space (the Montreal Neurological Institute [MNI] template; IXI549Space) using SPM12 unified segmentation and normalization procedure (Ashburner & Friston, 2005) with a reference functional image computed as the mean functional data after realignment across all timepoints omitting outlier scans. The output data were resampled to a common bounding box between MNI-space coordinates (−90, −126, −72) and (90, 90, 108), using 2 mm isotropic voxels and 4th order spline interpolation for the functional data, and 1 mm isotropic voxels and trilinear interpolation for the structural data. Last, the functional data were smoothed spatially using spatial convolution with a 4 mm FWHM Gaussian kernel.

#### 2.4.3 First-level analysis

Effects were estimated using a General Linear Model (GLM) in which each experimental condition was modeled with a boxcar function convolved with the canonical hemodynamic response function (HRF) (fixation was modeled implicitly, such that all timepoints that did not correspond to one of the conditions were assumed to correspond to a fixation period). Temporal autocorrelations in the BOLD signal timeseries were accounted for by a combination of high-pass filtering with a 128-second cutoff, and whitening using an AR(0.2) model (first-order autoregressive model linearized around the coefficient a = 0.2) to approximate the observed covariance of the functional data in the context of Restricted Maximum Likelihood estimation (ReML). In addition to experimental condition effects, the GLM design included first-order temporal derivatives for each condition (included to model variability in the HRF delays), as well as nuisance regressors to control for the effect of slow linear drifts, subject-motion parameters, and potential outlier scans on the BOLD signal.

### 2.5 Definition of functional regions of interest (fROIs)

Language and Multiple Demand (MD) fROIs were defined using a group-constrained subject-specific (GSS) approach (Fedorenko et al., 2010) where a set of spatial masks, or parcels, is combined with each individual subject’s localizer activation map, to constrain the definition of individual fROIs. The parcels delineate the expected gross locations of activations for a given contrast and are sufficiently large to encompass the variability in the locations of individual activations. Within each parcel, we selected the top 10% most localizer-responsive voxels, based on t-values.

To define the language fROIs, we used a set of five parcels derived from a group-level probabilistic activation overlap map for the *sentences* > *nonwords* contrast in 220 independent participants. The parcels included two regions in the left inferior frontal gyrus (LIFG, LIFGorb), one in the left middle frontal gyrus (LMFG), and two in the left temporal lobe (LAntTemp and LPostTemp). Following prior work (e.g., Blank et al., 2014), to define the right-hemisphere RH fROIs, the LH parcels were transposed onto the RH, but the individual LH and RH fROIs were allowed to differ in their precise locations within the homotopic parcels. Although the mask contained six parcels, we decided to exclude the left angular gyrus (LAngG) due to accumulating evidence that LAngG differs functionally from the rest of the core temporal and frontal areas and exhibits lower functional connectivity with those core areas (for discussion, see Shain et al., 2023). To define the MD fROIs, we used a set of 20 parcels (10 in each hemisphere) derived from a group-level probabilistic activation overlap map for the *hard* > *easy* spatial working memory contrast (Fedorenko et al., 2013) in 197 independent participants. The parcels included symmetrical regions in frontal and parietal lobes, as well as a region in the anterior cingulate cortex. All parcels are available for download from https://evlab.mit.edu/funcloc/.

### 2.6 Extraction of fMRI BOLD responses

We evaluated language and MD networks’ responses by estimating response magnitudes to the conditions of the standard and speeded language localizers in the individually defined fROIs. For each fROI in each participant, we averaged the responses across voxels to get a single value per participant per fROI per condition (i.e., the *sentences* and *nonwords* conditions for the language localizer tasks, and the *hard* and *easy* conditions for the MD localizer task). The responses to the conditions used to localize the areas of interest (e.g., the responses to the *sentences* and *nonwords* conditions in the language fROIs) were estimated using an across-runs cross-validation procedure, where one run of the standard or speeded language localizer was used to define the fROI and the other run of the same localizer version was used to estimate the response magnitudes. The procedure was repeated for each of the two run partitions, once where the first run was used for fROI definition and the second run was used for response estimation and once where the second run was used for fROI definition and the first run was used for response estimation. Finally, the estimates were averaged to derive a single value per participant per fROI per condition.

### 2.7 Statistical analysis

All statistical analyses were performed in RStudio (version 2021.09.2) or Python (version 3.9.2).

Linear mixed effects (LME) models were used to test for differences in BOLD response magnitudes and spatial correlations across localizer versions and conditions, while accounting for random effects from different participants and fROIs.

All LMEs reported in the paper were implemented in R using the *lmer* function from the **lme4** package (Bates et al., 2015; version 1.1-31). Fixed effects included the condition (*sentences* vs. *nonwords*) and the localizer version (standard vs. speeded). Their interaction was also included as a fixed effect where specified in order to test whether the effect of condition was different between the two localizer versions. Random intercepts were fit for participants and fROIs. Statistical significance testing was performed using the **lmerTest** package (Kuznetsova et al., 2017; version 3.1-3). Using this package, the t-statistic and associated p-values based on Satterthwaite’s approximation were computed. In cases where the interaction term was included, we compared the model with and without the *condition:version* interaction. Model comparison was performed using a likelihood ratio test implemented via the *anova* function in **lme4**, which yielded a $\chi^{2}$ test statistic.

For analyses involving a single dependent measure per condition (i.e., when comparing spatial correlations between activation maps across the two localizer versions), the fixed-effect included only the localizer version and random intercepts similarly included the participants and fROIs (e.g., *SpCorr LH language ~ version + (1|participant) + (1|fROI)*). Statistical significance was again computed using **lmerTest** based on the t-statistics when comparing model parameters.

For each LME reported, we provide tables containing the model formulae, fixed effects regression coefficients (β-values), t-values, p-values, and random effects coefficients (Supplementary 2E, 3D, 3E, 3F, 4B). R-squared values were computed using the *r.squaredGLMM* function from the **MuMIn** package (version 1.47.1).

In addition to LME analyses, pairwise comparisons between two conditions were conducted using two-sided paired or one-sample t-tests, as appropriate. These tests were implemented with the scipy.stats module in Python. All tests were two-tailed and statistical significance was assessed at α=0.05 .

## 3 Results

We compared the fMRI BOLD responses from two versions of a language localizer task (a ‘standard language localizer’ and a ‘speeded language localizer’). The results are organized according to the following two questions: (1) Can speeded reading be used to reliably localize language-responsive areas in individual participants?, and (2) Does increased processing difficulty during speeded reading affect brain responses in the domain-general Multiple Demand brain network?

### 3.1 The speeded language localizer can reliably localize language-responsive areas in individual participants

#### 3.1.1 The activation topography is similar between the standard and speeded language localizer versions

Twenty-four participants completed a standard language localizer task (Fedorenko et al., 2010) and a speeded localizer task. In both tasks, they silently read sentences (the *sentences* condition) and sequences of nonwords (the *nonwords* condition) (see Methods; fMRI tasks).

**Fig. 1.**
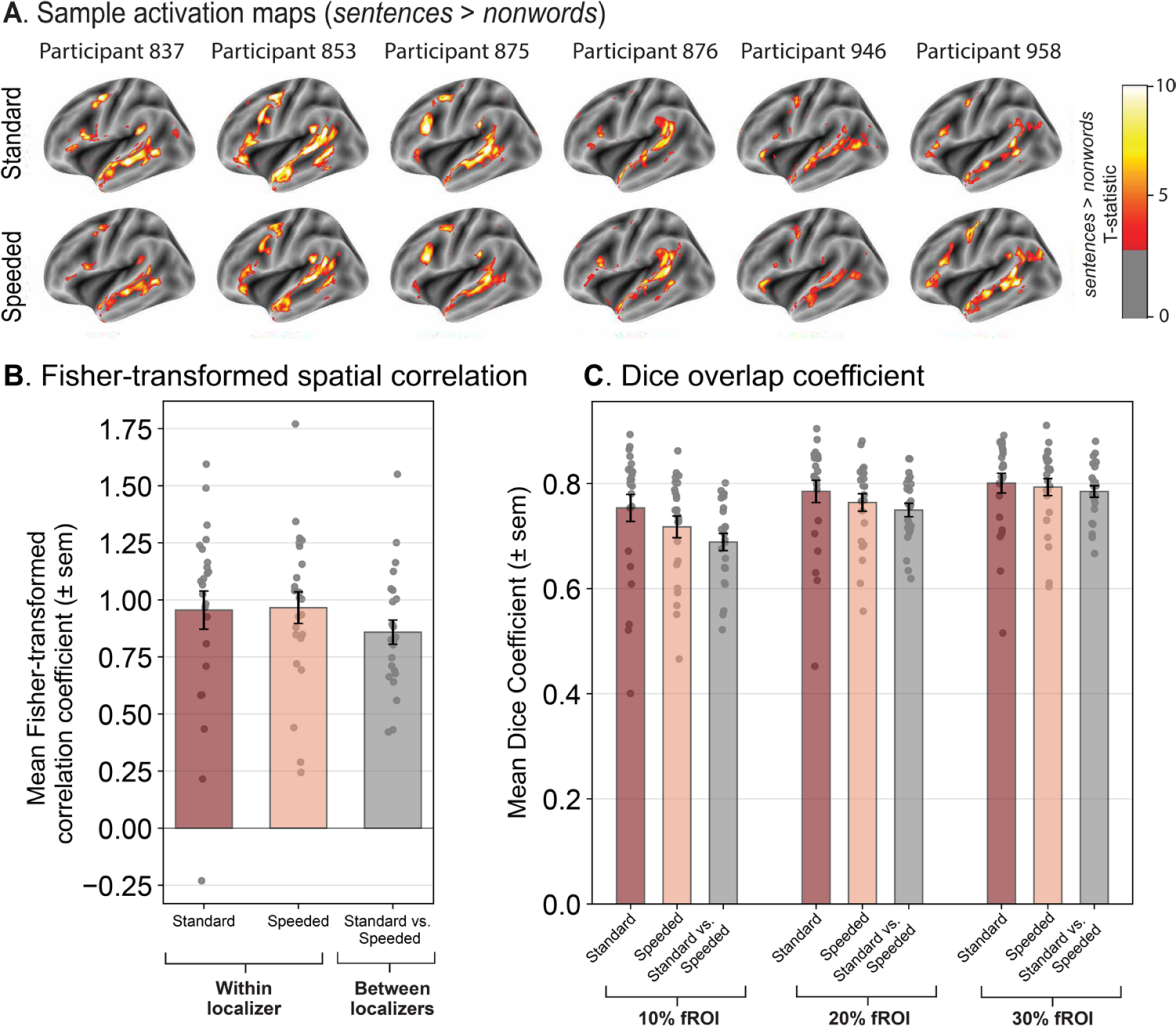
The activation topographies are highly similar between the standard and speeded language localizer versions. (A) Activation maps of the *sentences > nonwords* contrast in six sample participants for the standard language localizer (upper row) and the speeded language localizer (lower row). Activation maps are shown on the surface-inflated fsaverage template brain. The participant identifiers are numbers in the lab internal database and can be cross-referenced with the data tables on OSF. (B) The correlation of the voxel-wise activation patterns for the *sentences > nonwords* contrast within left-hemisphere (LH) language parcels (see Methods; Definition of fROIs) within and between localizer versions. The within localizer comparisons were performed by correlating the activation patterns between the two runs of the same localizer version (dark red bar = standard version; light red bar = speeded version; the data are averaged across participant and across five LH parcels within each participant), and the between localizer comparisons were performed by correlating the four pairwise combinations of runs between the two localizers (given two runs of each localizer; gray bar) and averaging them to obtain a single value. The dots correspond to the correlation values from individual participants (*n* = 24). Error bars show the standard error of the mean across participants. (C) The Dice overlap coefficient for the *sentences > nonwords* contrast computed between different runs of the same localizer version or between different runs across the two localizer versions. The fROIs were defined as the top 10%, 20%, or 30% of the most language-responsive voxels in the five LH language parcels. As in panel B, the two red bars show the Dice coefficients within a localizer version (between two runs of the same localizer; the data are averaged across participants and across five LH parcels within each participant) and the gray bar shows the Dice coefficient between localizer versions (averaging across four pairwise between-run comparisons). The dots correspond to the coefficient values from individual participants (*n* = 24). Error bars show the standard error of the mean across participants.

The activation maps for the *sentences > nonwords* contrast are visually highly similar between the standard and speeded language localizers (Fig. 1A; see Supplementary 2D for corresponding fROI surface maps in the same sample participants). To quantify this similarity, we correlated voxel-wise activation patterns (restricted to the LH language parcels; see Supplementary 2A for whole-brain correlations) across localizer runs and versions. The correlation values were Fisher-transformed and averaged across the five LH language parcels, leading to a single value for each comparison.

First, we correlated the activation patterns across the two runs *within* each localizer version. These values characterize the stability of the activation patterns for each version and also delimit the similarity that could be obtained between the two localizer versions. The within-localizer Fisher-transformed correlations were high for both versions: 0.955 and 0.966 for the standard and speeded versions, respectively (Fig. 1B; the two *within* bars on the left), and did not statistically differ from each other (*speeded* > *standard*; β = 0.011, t = 0.226, p = 0.821 via linear mixed effects (LME) modeling). Next and critically, we correlated the activation patterns between the two versions of the localizer. To match the amount of data to the within-version comparisons, we correlated activations for each run of the standard version with each run of the speeded version (four pairwise combinations, given two runs of each localizer version; Figure 1B). The between-localizer Fisher-transformed correlation coefficient was 0.859 (Fig. 1B; the *between* bar: the average of the four pairwise combinations of runs between the standard and speeded versions). To statistically compare the within- vs. between-version correlations, we modeled the average within-version and between-version correlation coefficients in an LME model with a fixed effect for comparison type (within vs. between), and random intercepts for participants and parcels. The similarity of the activations *within* a given localizer version was statistically higher than between localizer versions (Fig. 1B), with a relatively small effect size (Fig. 1B; *within > between*; β = 0.102, t = 2.763, p = 0.006), in line with both within and between correlations being high.

In a complementary analysis, we quantified the extent of voxel overlap between functional regions of interest (fROIs) using the Dice coefficient (Dice, 1945). The results mirrored the spatial correlation analyses above. The overlap between the *sentences > nonwords* fROIs, defined as the top 10% of language-responsive voxels, was high for both within and between comparisons (0.754 and 0.718 for the within comparisons for the standard and speeded versions, respectively; and 0.689 for the between comparison), but slightly higher across the runs within a localizer version than between localizer versions (β = 0.047, t = 3.291, p = 0.001) (Fig. 1C). For fROIs of larger size (e.g., fROIs defined as the top 20% or 30% of most language-responsive voxels within the parcels), the within vs. between differences get smaller (20%: β = 0.025, t = 2.281, p = 0.023; 30%: β = 0.012, t = 1.238, p = 0.217), which suggests that although the peaks of the activation topographies are slightly more similar within a localizer version than between the two versions, the overall topographies are highly similar (see Supplementary 2B for Dice coefficient comparisons across a larger range of fROI thresholds).

#### 3.1.2 The fROIs defined by the speeded language localizer respond at least as strongly and as selectively during language processing as the fROIs defined by the standard localizer

**Fig. 2.**
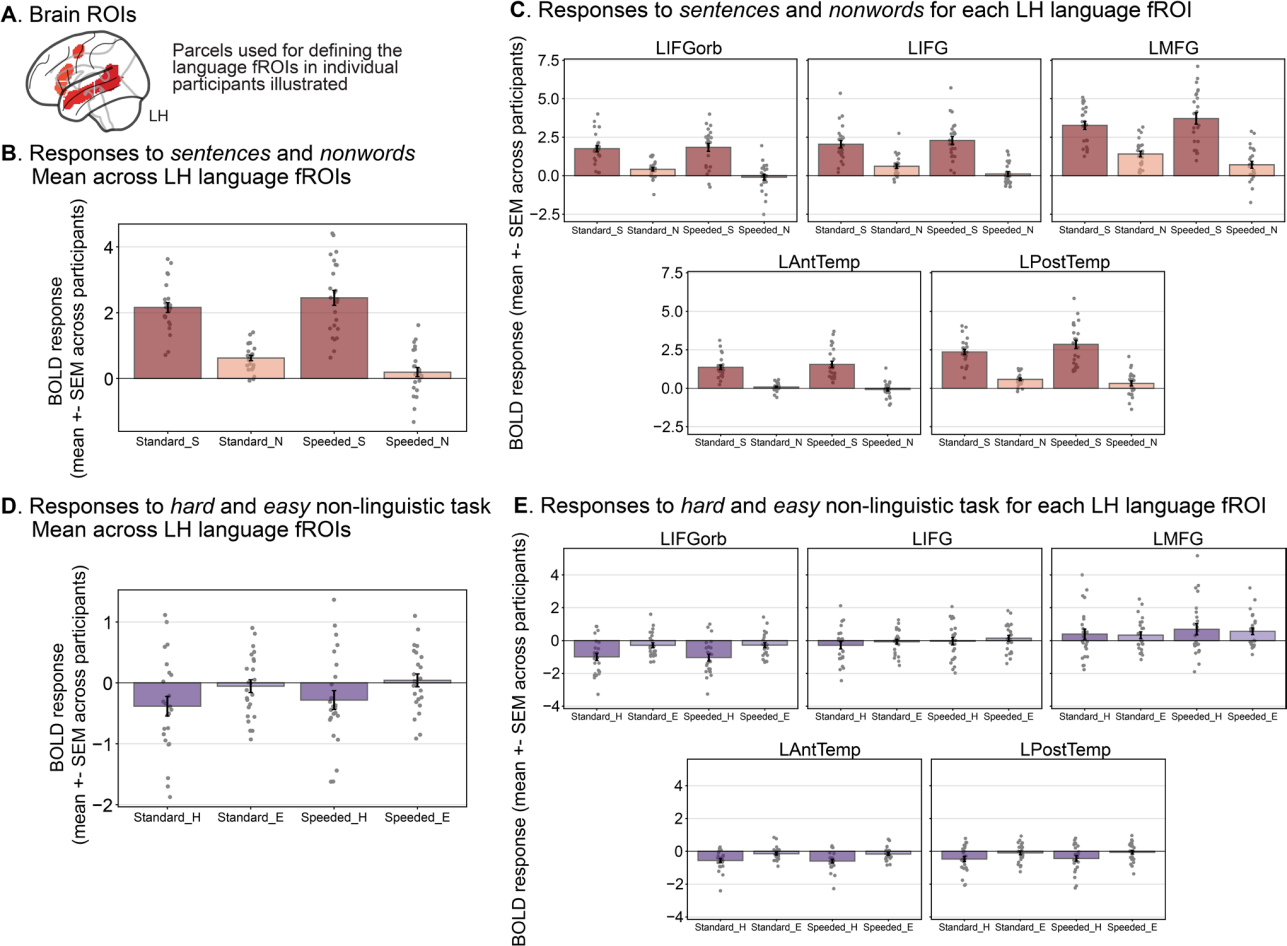
The speeded language localizer elicits a greater *sentences* > *nonwords* effect than the standard language localizer and the fROIs defined by the speeded localizer are similarly selective for language relative to a demanding non-linguistic task. (A) To define language fROIs, we used a set of masks (‘language parcels’; shown on the volumetric MNI152 template brain where all analyses were performed) within which most or all individuals in prior studies showed activity for the language localizer contrast in large samples (e.g., Fedorenko et al., 2010; Lipkin et al., 2022). We defined the LH language fROIs as the most language-responsive voxels (top 10%) within the borders of these five parcels for each participant and measured the BOLD response magnitude in these fROIs in a cross-validated manner (see Methods; Definition of fROIs). (B) Mean BOLD response to the language localizer conditions (S = *sentences*, N = *nonwords*) for the standard and speeded localizer versions averaged across the five LH language fROIs. (C) Mean BOLD response to the localizer conditions for each LH language fROI. (D) Mean BOLD response to a spatial working memory task consisting of two conditions, a *hard* condition (H) and an *easy* condition (E), averaged across the five LH language fROIs defined by the speeded and standard language localizers. See Supplementary 3A for evidence that the spatial working memory task elicited a robust *hard* > *easy* response in the MD network fROIs. (E) Mean BOLD response to the *hard* and *easy* spatial working memory task conditions for each LH language fROI.

Having established that the activation topographies are similar across the localizer versions (Fig. 1), we examined the magnitude of the BOLD responses for the *sentences* and *nonwords* conditions across the two versions in the fROIs defined by the standard approach of selecting top 10% of most language-responsive voxels within five broad, anatomical parcels (Fig. 2A; see Methods; Extraction of fMRI BOLD responses). Figure 2B shows the average BOLD responses across the five LH language fROIs and Figure 2C shows the responses for each of the five fROIs individually. To statistically compare the two localizers, the BOLD responses were modeled in an LME with fixed effects for condition (*sentences* vs. *nonwords*) and localizer version (standard vs. speeded) and random intercepts for participants and fROIs.

As expected, the effect of condition (estimated in independent data) was highly significant (*sentences > nonwords*, β = 1.897, t = 23.570, p < 0.0001) (Fig. 2B, C); in contrast, the main effect of localizer version was not significant (*speeded* > *standard*, β = -0.068, t = -0.851, p = 0.395). To further examine whether the standard and speeded versions differed with respect to their responses to *sentences* and *nonwords*, we used a similar LME as above but also included an interaction term between condition and localizer version. We tested for significance of the interaction using a likelihood-ratio test with a Chi Square test statistic ($\chi^{2}$). Indeed, the interaction was significant ($\chi^{2}$ = 20.653, p < 0.0001), suggesting that the responses to the two conditions (*sentences* and *nonwords*) differed between localizer versions. To better understand this difference, we examined the size of the *sentences* > *nonwords* contrast and found that it was greater in the speeded localizer compared to the standard localizer (*speeded > standard*; β = 0.723, t = 7.817, p < 0.0001). As can be seen in Figure 2B, C, the response to the *sentences* condition was higher in the speeded localizer compared to the standard localizer (*speeded > standard*; β = 0.293, t = 2.407, p = 0.017), and the response to the *nonwords* condition was lower in the speeded localizer compared to the standard localizer (*speeded > standard*; β = -0.430, t = -5.106, p < 0.0001). Taken together, these analyses show that the fROIs defined by the speeded language localizer show a larger *sentences* > *nonwords* effect compared to the standard localizer, due to both higher responses to *sentences* and lower responses to *nonwords* in the speeded version.

Because of the increasing interest in the field of language research in non-canonical language regions (e.g., Li et al., 2024; Tuckute et al., 2025; Wang et al., 2025), we also examined responses in the so-called *extended language network*, encompassing regions on the ventral temporal surface, in the medial frontal cortex, and in the cerebellum; Wolna et al., 2025). We found that the speeded localizer successfully identifies the regions of the extended language network and produces a similar response profile to the standard localizer (Supplementary 3G), demonstrating that the speeded version can be used to identify not only the core language areas but also non-canonical language-responsive areas. Following a reviewer’s request, we additionally examined responses to language in the Default Mode Network (DMN), which has been implicated in semantic processing (e.g., see Fernandino & Binder, 2024; cf. Humphreys et al., 2015). Using the same individual fROI approach as in our analyses of the language areas and using the *easy > hard* contrast from the spatial working memory task to define the DMN regions (following Mineroff, Blank et al., 2018), we did not find substantial responses to language in the DMN regions for either localizer version (with the exception of the left anterior temporal DMN region, which overlaps with the anterior temporal language parcel) (Supplementary 3H).

Next, we examined the selectivity of the language fROIs defined by both the standard and the speeded localizers for language processing relative to a non-linguistic demanding cognitive task. Prior work has established that language-responsive brain areas (as defined by standard versions of the language localizer) are highly selective for language relative to diverse non-linguistic inputs and tasks (e.g., Chen et al., 2023; Fedorenko et al., 2011; Ivanova et al., 2020, 2021; for reviews, see Fedorenko & Blank, 2020; Fedorenko et al., 2024). Here, we investigated whether the fROIs defined by the speeded language localizer exhibit a similar degree of selectivity. To do so, we collected brain responses during a spatial working memory task (see Methods; fMRI tasks) and examined BOLD response magnitudes to the *hard* and *easy* conditions in the LH language regions, defined by the standard versus speeded language localizers (Fig. 2D, E). As expected given the high topographic overlap between the two localizer versions reported in Section 1, both sets of fROIs showed selectivity for language, with no response during the cognitively demanding spatial working memory task (standard localizer: *hard*: t = -2.376, p = 0.026; *easy*: t = -0.505, p = 0.618 via two-sided, one-sample t-test against zero; speeded localizer: *hard*: t = -1.803, p = 0.085; *easy*: t = 0.397, p = 0.694). This lack of response in the language areas is in sharp contrast with the Multiple Demand areas, which respond strongly to both conditions, and show a clear *hard* > *easy* effect (Supplementary 3A).

Finally, in addition to the analyses reported in Sections 3.1.1 and 3.1.2 above, we tested whether the BOLD response magnitudes from the fROIs defined by the standard versus speeded localizers were stable over time (across runs (Supplementary 3B) and—for two participants who completed the localizers several times—across scanning sessions (Supplementary 3C)). This is important to know given that BOLD response magnitudes are often used in individual-differences investigations that aim to relate neural measures to behavior (e.g., Assem, Blank, et al., 2020; Kong et al., 2020; Mahowald & Fedorenko, 2016). We found that the magnitudes were indeed highly stable within participants over time.

In all panels, dots correspond to the responses of individual participants. Error bars show the standard error of the mean across participants. See Supplementary 3E for responses in the right-hemisphere (RH) language fROIs (which also show a reliable *sentences > nonwords* effect, similar to the LH fROIs, although the responses are overall weaker).

### 3.2 Speeded sentence reading engages the domain-general Multiple Demand (MD) system to a greater extent than standard reading

In addition to examining responses in the language network (Section 3.1.2), we investigated responses in the domain-general Multiple Demand (MD) network. This network supports computations related to goal-directed behaviors and is recruited during a broad array of cognitively demanding tasks (e.g., Assem, Glasser, et al., 2020; Duncan, 2010; Duncan et al., 2012, 2020; Fedorenko et al., 2013; Shashidhara et al., 2019). Of most relevance to the current investigation, the MD network appears to be engaged in some cases of effortful comprehension, including processing speech in noisy conditions or following acoustic degradation (Liu et al., 2022; MacGregor et al., 2022; Mattys & Wiget, 2011), processing accented speech (Adank & Janse, 2010; Adank et al., 2012; Banks et al., 2015; Janse & Adank, 2012), processing languages that one is not fully proficient in (Malik-Moraleda, Jouravlev et al., 2024; Wolna et al., 2024), and processing linguistic inputs that are not syntactically well-formed (Kauf et al., 2024; Kuperberg et al., 2003; Mollica et al., 2020; Nieuwland et al., 2012; Tuckute, Sathe, et al., 2024). However, understanding the full range of conditions under which the MD network is recruited during language processing remains an important research goal, and is critical for deciphering the nature of the MD network’s contributions to language.

**Fig. 3.**
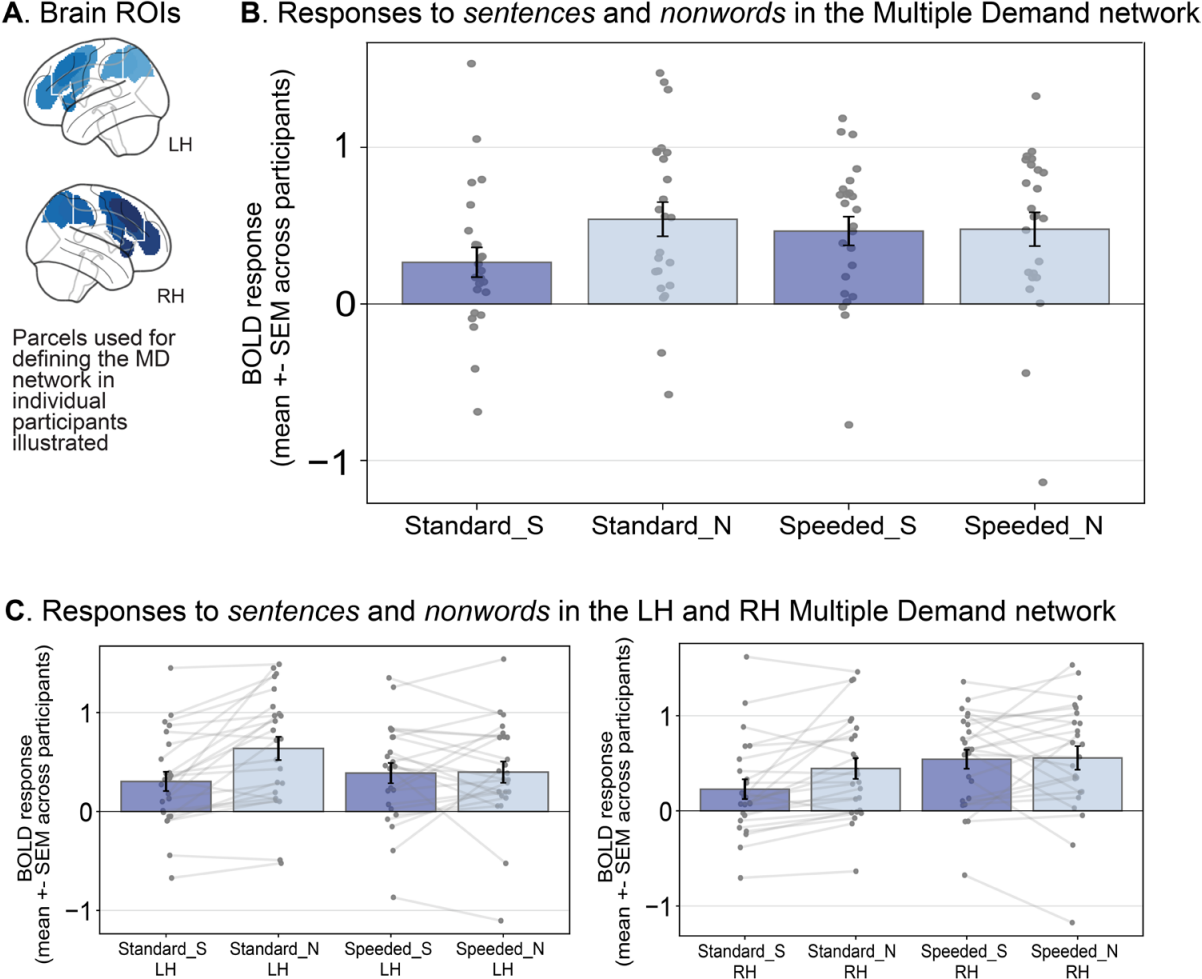
The Multiple Demand (MD) network is more engaged in speeded sentence reading compared to standard sentence reading. (A) To define MD fROIs, we used a set of masks (‘MD parcels’; shown on the volumetric MNI152 template brain) within which most or all individuals in prior studies showed activity for the MD *hard* > *easy* spatial working memory contrast in large samples (e.g., Diachek, Blank, Siegelman et al., 2020). We defined the LH and RH MD fROIs as the most working-memory-responsive voxels (top 10%) within the borders of the 20 parcels for each participant, and measured the BOLD response magnitude in these fROIs in a cross-validated manner (see Methods; Definition of fROIs). (B) Mean BOLD response to the language localizer conditions (S = *sentences*, N = *nonwords*) for the standard and speeded localizer versions averaged across the 20 LH/RH MD fROIs (see Supplementary 4A for individual fROIs). (C) Mean BOLD response to the language localizer conditions (S = *sentences*, N = *nonwords*) for the standard and speeded localizer versions averaged across the LH and RH MD fROIs. Light gray lines connect the responses to the two conditions for a given participant and localizer version.

Following prior work (e.g., Malik-Moraleda, Ayyash et al., 2022), we defined MD fROIs (10 in each hemisphere; Fig. 3A) using the *hard > easy* contrast of the spatial working memory task described in the previous section (Section 3.1.2; and Methods; fMRI tasks). We then examined the responses to the *sentences* and *nonwords* conditions across the two versions of the language localizer to test whether speeded reading taxes the MD network. (For validation that the MD fROIs behave as expected, that is, show a reliably greater response to the hard spatial working memory condition compared to the easy one, see Supplementary 3A.)

The BOLD response magnitudes for the *sentences* and *nonwords* conditions across both localizer versions are shown in Figure 3B for the average of the 10 left and right hemisphere MD fROIs and Figure 3C for each hemisphere separately (see Supplementary 4A for each of the 20 fROIs individually). In line with prior work (e.g., Diachek, Blank, Siegelman et al., 2020; Fedorenko et al., 2013), the MD fROIs showed a robust *nonwords* > *sentences* effect in the standard language localizer (*sentences > nonwords*, β = -0.275, t = -7.547, p < 0.0001). In contrast, in the speeded version, reading of *nonwords* did not engage the MD network to a greater extent than reading of *sentences* (*sentences* > *nonwords*, β = -0.012, t = -0.283, p = 0.777). As evident from Figure 3C, some participants exhibited higher MD network engagement in the *nonwords* condition, whereas others exhibited the opposite pattern. To statistically compare the responses to the two localizers, the BOLD responses were modeled in an LME with fixed effects for condition (*sentences* vs. *nonwords*) and localizer version (standard vs. speeded) and random intercepts for participants and fROIs. Using likelihood ratio tests, we confirmed a significant interaction between condition and localizer version ($\chi^{2}$ = 18.274, p < 0.0001), suggesting that the MD network was engaged differently by the two localizers. In particular, the MD network was more engaged in the *sentences* condition during the speeded localizer compared to the standard localizer (*speeded* > *standard*, β = 0.200, t = 4.584, p < 0.0001), whereas the responses to the *nonwords* condition did not reliably differ between the two versions (*speeded > standard*, β = -0.064, t = -1.519, p = 0.129). In summary, speeded sentence reading was more effortful than slower-paced reading, and under the speeded-reading conditions, no *nonwords > sentences* effect was observed.

In all panels, dots correspond to the responses of individual participants. Error bars show the standard error of the mean across participants.

## 4 Discussion

In cognitive neuroscience, there is a growing recognition of inter-individual differences in the precise functional topographies, especially in the association cortex (e.g., Braga et al., 2020; DiNicola et al., 2023; Du et al., 2024; Frost & Goebel, 2012; Gratton & Braga, 2021; Tahmasebi et al., 2012). We here show that a standard localizer for the language network (Fedorenko et al., 2010) can be halved in time by using speeded reading, and that the speeded-reading-based contrast is even more robust than the one based on standard-paced reading. In the remainder of the Discussion, we elaborate on these findings and their implications.

### 4.1 Robustness and generalizability of the language localizer

The standard language localizer (Fedorenko et al., 2010) investigated in our study has been widely used over the past decade (e.g., Braga et al., 2020; Du et al., 2024; Fedorenko et al., 2010; Lipkin et al., 2022; Mahowald & Fedorenko, 2016). The localizer contrasts the reading of well-formed sentences versus sequences of nonwords. The brain areas identified by this contrast have been shown to be robust across different linguistic materials (e.g., Fedorenko et al., 2010)—an effect we replicate here—and tasks (e.g., Diachek, Blank, Siegelman et al., 2020; Gao et al., 2025). Moreover, this contrast generalizes to the auditory and audio-visual presentation modalities (e.g., Fedorenko et al., 2010; Olson et al., 2023; Scott et al., 2017) and works across typologically diverse languages (Malik-Moraleda et al., 2022; Richardson et al., 2020; Terhune-Cotter, 2025) and for diverse populations, including children (Hiersche et al., 2024; Ozernov-Palchik et al., 2024), older healthy adults (Billot et al., 2024), and individuals with stroke aphasia (Billot et al., 2026; Clercq et al., 2024). In the current study, we show that the reading version of the localizer is robust to presentation speed, in line with past behavioral work showing the ability to understand language at fast speeds when presented word-by-word in a rapid serial visual presentation (RSVP) paradigm (e.g., Forster, 1970; Mollica & Piantadosi, 2017; Potter, 2012; Potter et al., 1980, 1986), and in line with prior group-averaging fMRI studies observing activation in canonical language areas for speeded reading (Benjamin & Gaab, 2012; Vagharchakian et al., 2012). In the speeded version that we evaluated, each word was presented for 200 ms (compared to 450 ms in the standard localizer, i.e., ~56% faster), and we demonstrate that language areas in individual participants can be reliably localized using this version.

### 4.2 The speeded language localizer shows at least as strong selectivity for language relative to the control condition and a non-linguistic demanding task

In the current work, we first established that the voxel-level activation topographies were highly similar between the standard and speeded language localizers, and then demonstrated that the response magnitudes in fROIs defined by each localizer version were also similar both in their responses to language and a control condition, and in their selectivity for language relative to a non-linguistic working memory task (e.g., Duncan, 2010; Fedorenko et al., 2013). Moreover, the speeded localizer is actually more effective than the standard version given that it better differentiates the critical language condition and the control condition. Specifically, the speeded localizer elicited a stronger response to the *sentences* condition, possibly due to an increase in attentional demands or processing difficulty (but see next discussion section), and a weaker response to the control condition (*nonwords*). The reduced response to nonwords may be due to the increased challenge of reading nonwords quickly which in turn might reduce the accessibility of information about their phonotactic properties (e.g., Regev et al., 2024). Thus, the speeded localizer produced at least as strong a response to language as the standard localizer. Additionally, the areas identified by the speeded localizer were selective for language relative to a non-linguistic spatial working memory task, similar to the profile of the areas identified using the standard localizer (see Fedorenko & Blank, 2020 and Fedorenko et al., 2024 for reviews). Finally, the size of the *sentences > nonwords* contrast was stable across runs, which suggests that the speeded localizer can also be used in studies that relate neural markers to behavior or genetics to study individual differences (e.g., Assem, Blank, et al., 2020; Mahowald & Fedorenko, 2016).

### 4.3 Contributions of the Multiple Demand (MD) network to language comprehension

The Multiple Demand (MD) network is engaged during diverse cognitively demanding tasks, including classic executive function tasks (Assem, Glasser, et al., 2020; Duncan, 2010; Duncan & Owen, 2000; Duncan et al., 2012, 2020; Fedorenko et al., 2013; Shashidhara et al., 2019), and has been linked with constructs such as attention, working memory, and cognitive control. The contributions of this network to language processing remain an active area of investigation. So far, three key findings have emerged.

***First***, language paradigms where comprehension/production is accompanied by extraneous task demands—for example, performing meta-linguistic judgments or answering comprehension questions—engage the MD network, in addition to the language network (e.g., Diachek, Blank, Siegelman et al., 2020; Gao et al., 2025; see Christodoulou et al., 2014; Cutting et al., 2006, *inter alia*, for earlier, indirect evidence). ***Second***, during passive auditory comprehension, *linguistic* demands (e.g., the costs of retrieving an infrequent word, or forming a non-local syntactic dependency) modulate activity in the language-selective system, not the MD network (Shain et al., 2022; Wehbe et al., 2021; for a review, see Fedorenko & Shain, 2021). And ***third***, some cases of effortful comprehension—even absent external task demands—engage the MD network. Such cases include listening to speech in noisy conditions (Liu et al., 2022; MacGregor et al., 2022; Mattys & Wiget, 2011; Wild et al., 2012) and in quiet conditions for cochlear implant users (Sherafati et al., 2022), processing accented speech (Adank & Janse, 2010; Adank et al., 2012; Banks et al., 2015; Janse & Adank, 2012), processing a language that one is not fully proficient in (Malik-Moraleda, Jouravlev et al., 2024; Wolna et al., 2024), and processing linguistic inputs that are not syntactically well-formed (Kauf et al., 2024; Mollica et al., 2020; Tuckute, Sathe, et al., 2024). Interestingly, passive reading, even at typical speeds and in proficient readers, sometimes elicits an above-baseline response in the MD network, in contrast to passive listening (e.g., Gao et al., 2025; current study), which suggests that reading—a relatively late-acquired skill—may be associated with some amount of cognitive effort. Speeded reading requires more effort, leading to a 43% higher response to the sentences condition compared to the standard-speed version in the MD network (for earlier evidence, see Vagharchakian et al., 2012, although the evidence is indirect as no independent MD localizer is included). The nature of the MD network’s contributions during different kinds of effortful linguistic processing, and whether these contributions are causally important (e.g., MacGregor et al., 2022), remains to be determined.

One practical corollary of the fact that for the speeded version, the responses in the MD network are similar in magnitude for the sentence and nonword reading conditions, is that the reverse, *nonwords > sentences* contrast cannot be used to localize the MD network, in contrast to the standard language localizer (Diachek, Blank, Siegelman et al., 2020; Fedorenko et al., 2013; Shain et al., 2020). Whether the time saved by the speeded language localizer version is worth this trade-off of not being able to functionally define the MD regions using the same localizer will depend on the researcher’s goals.

### 4.4 Other efforts in cognitive neuroscience to develop efficient localizers

Functional localizers increase the sensitivity, functional resolution, and interpretability of research in cognitive neuroscience, but they take up precious time during the study. As a result, there is growing interest in making localizers more efficient. Two approaches have been taken: i) reducing the number and/or the duration of experimental blocks, or ii) trying to optimize the critical stimuli so as to increase the size of the *critical > control* effect. Our approach falls into the first category: by increasing the speed of (visually) presenting linguistic materials (by ~56%), we shortened experimental blocks from 18 seconds (3 6-second trials) to 9 seconds (3 3-second trials). (Note that although we retained the original 14 seconds fixation blocks for maximum comparability with the standard version, the fixation blocks could likely be shortened to 9 seconds, which would shave off another 30 seconds from the run’s duration.) Lee et al. (2024) also took the first approach, but instead of changing the presentation speed, they iteratively removed blocks and examined the consequences on brain responses. They showed that for a standard auditory language localizer based on the contrast of *intact speech > degraded speech* (as introduced in Scott et al., 2017) reducing the two-run protocol from ~12 minutes (32 blocks total, 16 per condition) to ~6 minutes (8 blocks per condition) and even ~3.5 minutes (4 blocks per condition) does not compromise the ability to localize the language regions.

The approach of stimulus optimization was used by Dodell-Feder et al. (2011), who analyzed responses to individual stimuli in a standard Theory of Mind (ToM) network localizer (Saxe & Kanwisher, 2003). Using a large dataset of a few hundred participants, they identified a) a subset of the critical-condition items (false belief stories) that elicit the highest response in the ToM brain areas, and b) a subset of the control-condition items (false photograph stories) that elicit the lowest response in the ToM areas. These subsets were used to create a highly efficient ToM localizer (see Chen, Kamps et al., 2024 for a related approach). Other studies have attempted to select stimuli that would be especially exciting for particular individuals based on their interests. For example, Olson et al. (2024) used language materials on topics of interest to different individuals with autism and found stronger responses in the language areas with those custom-selected stimuli. Finally, with the advent of neural networks that are predictive of brain responses (e.g., Schrimpf et al., 2021; Tuckute, Kanwisher, et al., 2024; Yamins et al., 2014), it is now possible to create or select stimuli that elicit maximal responses in the target region/network (Bashivan et al., 2019; Gu et al., 2023; Ratan Murty et al., 2021; Tuckute, Sathe, et al., 2024; Xiao & Kreiman, 2020). To our knowledge, these advances have not yet been leveraged in the creation of efficient localizers, but they certainly can and should be.

In addition to increasing the efficiency of a given localizer, another recent effort is to combine several localizers into a single experiment. For example, Marvi, Hutchinson et al. (2025) propose a multimodal localizer with simultaneous presentation of visual stimuli, such as faces, bodies, and scenes, which can be processed relatively automatically (e.g., Bugatus et al., 2017; Mur et al., 2025), and auditory stimuli, such as meaningless speech or short passages, which participants are instructed to focus on. This multimodal localizer elicits brain responses comparable to administering all the different contrasts as independent tasks, leading to a huge efficiency gain.

Increasing localizer efficiency in all these ways is valuable given the increasing popularity of precision imaging approaches in cognitive neuroscience (Allen et al., 2022; Gordon et al., 2017; Gratton & Braga, 2021; Naselaris et al., 2021).

### 4.5 Limitations

A limitation of the speeded localizer is that it requires a certain level of reading proficiency. We took inspiration from prior behavioral evidence of successful linguistic processing in rapid serial visual presentation (RSVP) paradigms at rates that are even faster than the 200 ms/word used here (e.g., Forster, 1970; Mollica & Piantadosi, 2017; Potter, 2012; Potter et al., 1980, 1986). But those earlier studies used proficient young adult readers. The speeded localizer may be less suitable for children, older adults, and other populations that may have difficulties with the demands of reading at fast rates or simply with quickly flashing words (e.g., Christodoulou et al., 2014).

A limitation of the current study design is that the standard language localizer was always administered prior to the speeded version (typically separated by other, unrelated tasks), so differences, or lack thereof, between the two versions could be confounded by task order. Although the experimental materials were always different between the versions, repeating a similar task within a session can modulate BOLD responses through practice with the task, practice-related changes in strategy, or reduced attention later in the session. However, these kinds of confounds should lead to lower BOLD responses later in the session (e.g., Grill-Spector et al., 2006; Landau et al., 2004). However, we observe that the response to the critical sentence condition in the speeded version is actually *higher* than in the standard version despite being administered later in the session. It is possible that doing the reading in an RSVP paradigm at a slower rate makes it easier to perform it at a faster rate later, so performing the speeded version first may lead to an even stronger response to the sentence condition, but this possibility remains to be evaluated.

### 4.6 Conclusions

We hope that researchers working with populations who are proficient readers would benefit from this version of the language localizer, and that creating more efficient localizers, like this one, would lead to an even more widespread adoption of functional localization as a way to build a cumulative research enterprise where findings can be more straightforwardly compared across studies and labs.

## Supplementary Material

Supplementary Material

## Data Availability

The scripts for running the speeded language localizer as well as the associated analyses can be found here: https://github.com/el849/speeded_language_localizer/. The data can be found on OSF: https://osf.io/2vskh/.
