# Supplementary material for: A 3.5-minute-long reading-based fMRI localizer for the language network
Source: Imaging Neurosci (Camb). 2026 May 26;4:IMAG.a.1246. doi: 10.1162/IMAG.a.1246 (PMC13214572; doi:10.1162/IMAG.a.1246)
Supplement: Supplementary Material [file IMAG.a.1246_supp.pdf]

Supplementary Information:

A 3.5-minute-long reading-based fMRI localizer for the language network

Greta Tuckute* , Elizabeth Jiachen Lee*, Aalok Sathe, Evelina Fedorenko

Overview

A 3.5-minute-long reading-based fMRI localizer for the language network 1
SI 1: Details on fMRI acquisition sequences 2
SI 2: Information related to Results Section 3.1.1 3
SI 3: Information related to Results Section 3.1.2 8
SI 4: Information related to Results Section 3.2 (Multiple Demand network) 15
Supplemental Information References 17

SI 1: Details on fMRI acquisition sequences

	A	B	C
**Number Participants**	22*	2*	1
**Sequence**			
**Type**	SMS-EPI	EPI with GRAPPA	EPI with GRAPPA
**Flip Angle**	90	90	90
**Acceleration Factor**	2	2	2
**Acquisition Parameters**			
**Number Slices**	52	33	31
**Slice Thickness**	2mm	4mm	4mm
**In-plane Resolution**	2mm x 2mm	2.1mm x 2.1mm	2.1mm x 2.1mm
**FoV**	208mm	200mm	200mm
**FoV Matrix Size**	104 x 104	96 x 96	96 x 96
**TR**	2000ms	2000ms	2000ms
**TE**	30ms	30ms	30ms

SI Table 1. **Functional MRI acquisition sequences.**
* One participant had the standard language localizer acquired using sequence B, and the speeded language localizer acquired using sequence A. For all remaining participants, the acquisition sequence was kept constant in all comparisons between the standard and the speeded versions of the language localizer. For acquisition of the Multiple Demand (MD) localizer task, all participants besides one, completed the MD localizer task in the same session as the language localizer tasks. The remaining participant completed the two language localizer tasks using sequence B, while the MD task was acquired using sequence A in a later session.

SI 2: Information related to Results Section 3.1.1

SI 2A: Whole-brain spatial correlation (supplementing language parcel correlations in Figure 1B and Figure 1C)

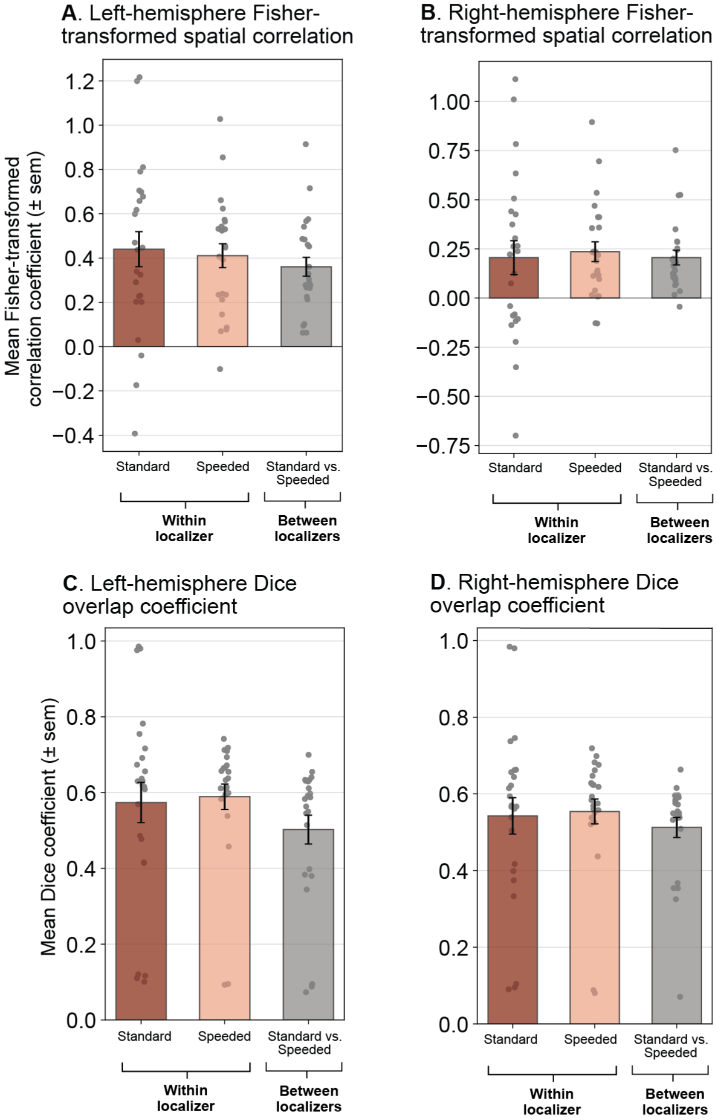

SI Figure 2A: Correlation of whole-brain voxel-wise activation patterns and overlap coefficient within and between language localizer versions (for the left and right hemispheres separately).

(A-B) We quantified the correlation of the voxel-wise activation patterns for the *sentences* > *nonwords* contrast within the whole left hemisphere (**panel A**) and right hemisphere (**panel B**) within localizer versions (between the two runs of the same localizer; red bars) and between localizer versions (for a total of four such pairwise combinations, given two runs of each localizer version; gray bar). In both panels, the bars show the average Fisher-transformed correlation coefficient across participants and individual points show the correlation values from individual participants (n=24). Error bars show the standard error of the mean across participants.

(C-D) For an additional metric of the similarity of voxel-wise activation patterns for the *sentences* > *nonwords* contrast between the standard and speeded localizers, we computed the Dice coefficient overlap

within the whole left hemisphere (**panel C**) and right hemisphere (**panel D**) within localizer versions (between the two runs of the same localizer; red bars) and between localizer versions (for a total of four such pairwise combinations, given two runs of each localizer version; gray bar). The Dice coefficient was computed as: $2 * |Standard \cap Speeded| / (|Standard| + |Speeded|)$ for each hemisphere, where $|Standard \cap Speeded|$ denotes the number of voxels that were in the top 10% responsive voxels for both the standard and speeded localizer versions, $|Standard|$ denotes the number of voxels in the top *10%* for the standard localizer version, and $|Speeded|$ denotes the number of voxels in the top 10% for the speeded localizer version (Note: $|Standard| = |Speeded|$ because the same parcels were used for both localizer versions). This computation provides a value between 0 and 1, where 0 indicates that the two localizer versions identified completely non-overlapping regions, and 1 indicates that the two localizer versions identified completely overlapping regions. In both panels, the bars show the average Dice coefficient across participants and individual points show the overlap values from individual participants (*n*=24). Error bars show the standard error of the mean across participants.

SI 2B: Dice overlap coefficient between the standard and speeded language localizer versions

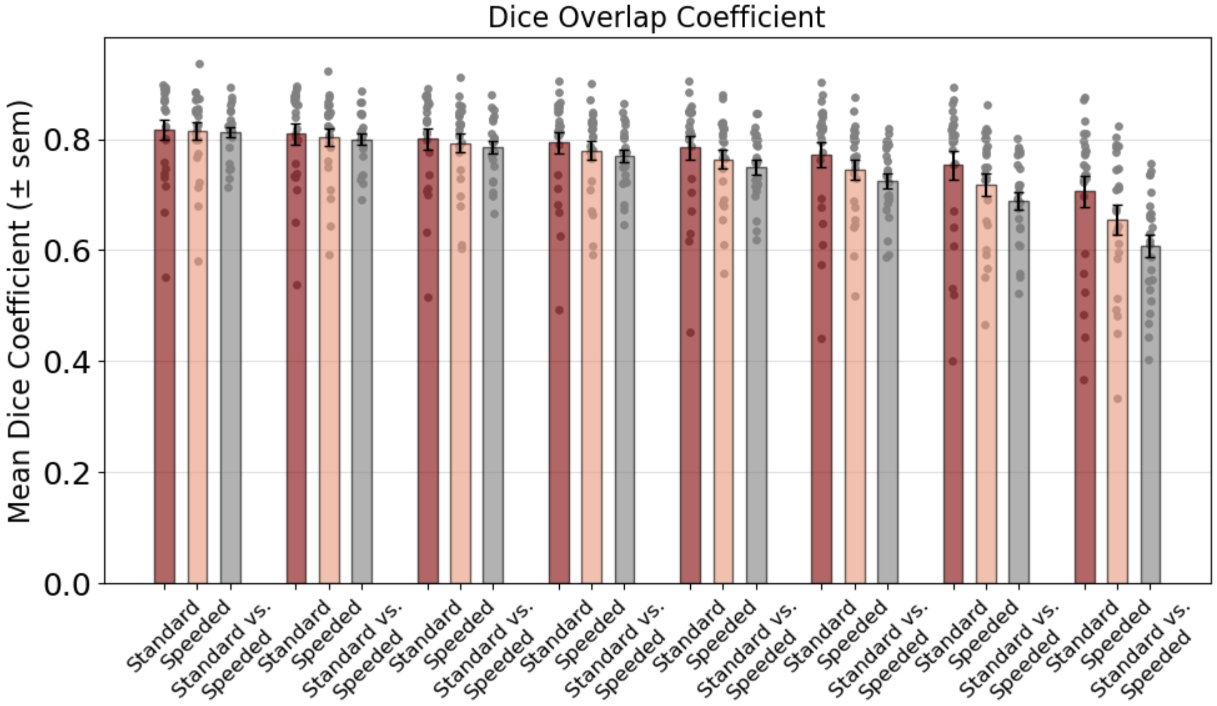

SI Figure 2B. Dice coefficient overlap values across a range of fROI definition thresholds.
We quantified the Dice coefficient overlap at a range of fROI definition thresholds. For each participant, the top *n*% most responsive voxels to the *sentences* > *nonwords* contrast in each of the LH language parcels were selected in both the standard and speeded localizer versions. *n* denotes the percentage threshold for fROI inclusion, and we show results for *n* = [5, 10, 15, 20, 25, 30, 35, 40]. The bars above show the average Dice coefficient across the five LH language fROIs.
Note that in some participants (in particular for larger values of *n*), not all top *n*% voxels displayed a positive *sentences* > *nonwords* t-statistic. In this case, the voxels with negative t-statistic (i.e., opposite selectivity) were excluded from the Dice coefficient analyses. See the number of included voxels across the range of *n* in SI Table 2C.

			LH_IFGorb		LH_IFG		LH_MFG		LH_AntTemp		LH_PostTemp	
Firstlevel	Constant	Threshold (top n%)	Mean	StDev	Mean	StDev	Mean	StDev	Mean	StDev	Mean	StDev
Standard	S-N	40	145.167	9.412	276.958	67.322	181.875	14.069	651.000	0.000	1179.000	0.000
		35	128.750	4.484	243.708	57.326	160.083	9.390	570.000	0.000	1032.000	0.000
		30	111.333	1.857	209.792	47.352	138.042	4.695	488.000	0.000	885.000	0.000
		25	93.708	3.470	175.875	37.635	116.000	0.000	407.000	0.000	737.000	0.000
		20	75.500	7.348	141.958	28.440	93.000	0.000	326.000	0.000	590.000	0.000
		15	56.000	0.000	107.833	20.412	70.000	0.000	244.000	0.000	443.000	0.000
		10	37.000	0.000	72.375	12.860	47.000	0.000	163.000	0.000	295.000	0.000
		5	19.000	0.000	36.917	5.307	24.000	0.000	82.000	0.000	148.000	0.000
	ODD_S-N	40	143.750	9.918	273.125	65.409	181.125	11.066	651.000	0.000	1179.000	0.000
		35	129.292	5.137	242.958	54.865	160.792	4.303	570.000	0.000	1032.000	0.000
		30	112.208	6.359	210.708	45.028	139.000	0.000	488.000	0.000	885.000	0.000
		25	93.000	0.000	178.083	36.710	116.000	0.000	407.000	0.000	737.000	0.000
		20	74.000	0.000	143.042	29.190	93.000	0.000	326.000	0.000	590.000	0.000
		15	56.000	0.000	107.583	21.637	70.000	0.000	244.000	0.000	443.000	0.000
		10	37.000	0.000	72.125	14.085	47.000	0.000	163.000	0.000	295.000	0.000
		5	19.000	0.000	36.667	6.532	24.000	0.000	82.000	0.000	148.000	0.000
	EVEN_S-N	40	137.292	25.752	268.500	70.902	174.958	33.146	651.000	0.000	1163.458	47.110
		35	122.417	19.525	237.667	58.238	154.125	26.713	570.000	0.000	1030.417	5.563
		30	106.292	13.343	206.833	46.400	133.042	20.230	488.000	0.000	885.000	0.000
		25	90.542	7.763	174.917	35.466	111.958	13.757	407.000	0.000	737.000	0.000
		20	73.750	3.926	141.792	25.307	90.875	7.321	326.000	0.000	590.000	0.000
		15	57.250	5.712	107.875	15.470	69.708	1.429	244.000	0.000	443.000	0.000
		10	37.000	0.000	73.542	7.144	47.000	0.000	163.000	0.000	295.000	0.000
		5	19.000	0.000	38.000	0.000	24.000	0.000	82.000	0.000	148.000	0.000
Speeded	S-N	40	148.000	0.000	294.625	8.272	185.000	0.000	651.000	0.000	1179.000	0.000
		35	130.000	0.000	260.000	0.000	162.000	0.000	570.000	0.000	1032.000	0.000
		30	111.000	0.000	223.000	0.000	139.000	0.000	488.000	0.000	885.000	0.000
		25	93.000	0.000	186.000	0.000	116.000	0.000	407.000	0.000	737.000	0.000
		20	74.000	0.000	149.000	0.000	93.000	0.000	326.000	0.000	590.000	0.000
		15	56.000	0.000	112.000	0.000	70.000	0.000	244.042	0.204	443.000	0.000
		10	37.000	0.000	75.000	0.000	47.000	0.000	163.000	0.000	295.000	0.000
		5	19.000	0.000	38.000	0.000	24.000	0.000	82.000	0.000	148.000	0.000
	ODD_S-N	40	148.000	0.000	286.708	38.150	185.000	0.000	651.000	0.000	1179.000	0.000
		35	130.000	0.000	252.792	28.915	162.000	0.000	570.000	0.000	1032.000	0.000
		30	111.000	0.000	218.750	20.821	139.000	0.000	488.000	0.000	885.000	0.000
		25	93.000	0.000	183.292	13.268	116.000	0.000	407.000	0.000	737.000	0.000
		20	74.000	0.000	147.833	5.715	93.000	0.000	326.000	0.000	590.000	0.000
		15	56.000	0.000	112.000	0.000	70.000	0.000	244.000	0.000	443.000	0.000
		10	37.000	0.000	75.000	0.000	47.000	0.000	163.000	0.000	295.000	0.000
		5	19.000	0.000	38.000	0.000	24.000	0.000	82.000	0.000	148.000	0.000
	EVEN_S-N	40	141.417	24.702	289.875	25.197	185.000	0.000	646.625	21.433	1168.333	52.256
		35	124.917	20.231	255.958	15.482	162.000	0.000	569.000	4.899	1027.458	22.250
		30	107.500	15.916	221.500	7.348	139.000	0.000	488.000	0.000	885.000	0.000
		25	90.500	12.247	186.000	0.000	116.000	0.000	407.000	0.000	737.000	0.000
		20	72.292	8.369	149.000	0.000	93.000	0.000	326.000	0.000	590.000	0.000
		15	55.042	4.695	112.000	0.000	70.000	0.000	244.000	0.000	443.000	0.000
		10	36.833	0.816	75.000	0.000	47.000	0.000	163.000	0.000	295.000	0.000
		5	19.000	0.000	38.000	0.000	24.000	0.000	82.000	0.000	148.000	0.000

SI Table 2C. Number of included voxels across fROI definition thresholds for Dice overlap analyses. Mean and standard deviation for the number of voxels across participants in each left-hemisphere language fROI that were included in the fROIs for the Dice coefficient overlap analyses (i.e., voxels with a positive t-value corresponding to the *sentences* > *nonwords* contrast). For the Dice analyses, voxels that demonstrate the opposite selectivity (negative t-values) were excluded. The total number of voxels in the parcels were: LH_IFGorb: 370; LH_IFG: 743, LH_MFG: 462, LH_AntTemp: 1627, LH_PostTemp: 2948, and if no participants displayed negative t-values for the *sentences* > *nonwords* contrast the number of voxels included in the Dice analyses would always correspond to a given percentage threshold (*n*). As evidenced from the table, in most cases all *n* % voxels show positive t-values, but occasionally some voxels are excluded (in particular, for larger *n*).

SI 2D: Sample participant fROI Maps

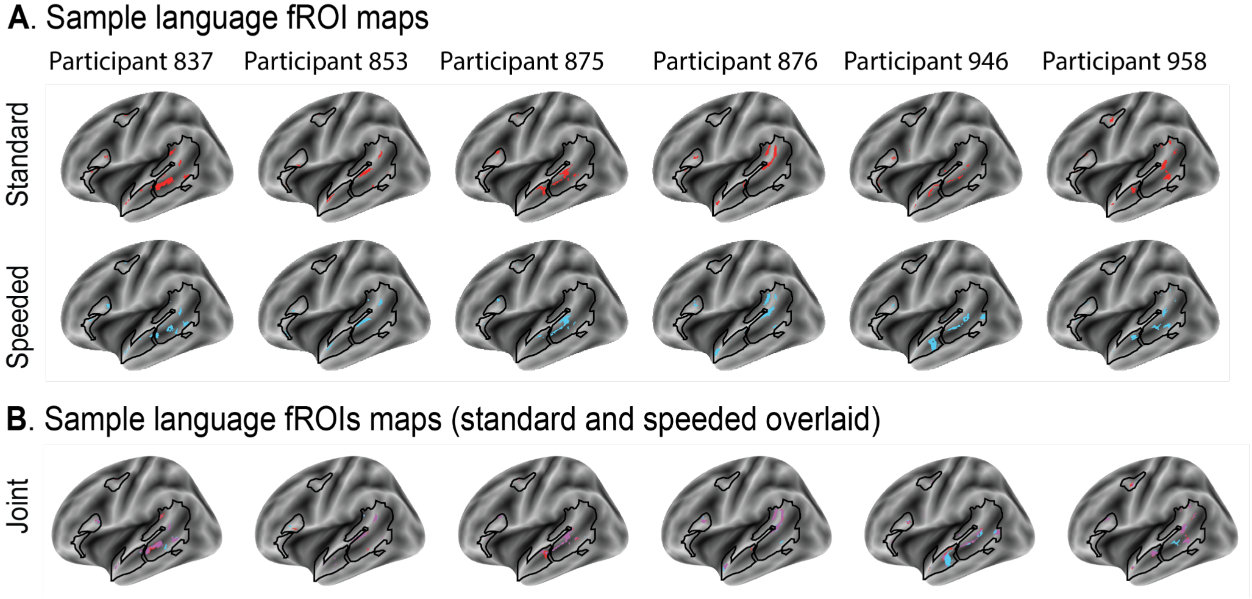

SI Figure 2D. fROI maps for the standard and speeded language localizers.
(A) Binary maps of the language fROIs in six sample participants for the standard language localizer (upper row in red) and the speeded language localizer (lower row in blue). fROIs were defined by selecting the top 10% most responsive voxels to the *sentences* > *nonwords* contrast (see Methods; Definition of fROIs). Maps are shown on the surface-inflated fsaverage template brain. The participant identifiers are numbers in the lab internal database and can be cross-referenced with the data tables on OSF.
(B) Overlay of the language fROI maps from **(A)**. The fROI defined using the standard language localizer is shown in red, the fROI defined using the speeded language localizer is shown in blue. Regions that are overlapping in the two localizers are shown in purple.

SI 2E: Statistics tables for Results Section 3.1.1

In the tables below, "SpCorr" denotes the Fisher-transformed spatial correlation coefficient. "within_between" denotes whether a spatial correlation coefficient was computed within localizer or between localizer versions. "participant" denotes each of the *n*=24 participants. "fROI" denotes each of the LH or RH regions of interest (five fROIs in each hemisphere).

SpCorr LH language ~ version + (1|participant) + (1|fROI)

Spcorr comparison of (within) standard and (within) speeded localizers, $r^2 = 0.4024$

	Estimate	Std. Error	df	t value	Pr(>|t|)
(Intercept)	0.955	0.078	22.310	12.221	0.000
VersionSpeeded	0.011	0.047	211.788	0.226	0.821

SpCorr LH language ~ within_between + (1|participant) + (1|fROI)

Spcorr within localizer vs. between localizer versions, $r^2 = 0.432$

	Estimate	Std. Error	df	t value	Pr(>|t|)
(Intercept)	0.859	0.074	24.566	11.601	0.000
Conditionwithin	0.102	0.037	331.871	2.763	0.006

SpCorr language (LH wholebrain) ~ within_between + (1|participant)

Spcorr within localizer vs. between localizer versions (left-hemisphere wholebrain), r^2 = 0.5399

	Estimate	Std. Error	df	t value	Pr(>|t|)
(Intercept)	0.360	0.059	45.810	6.076	0.000
Conditionwithin	0.065	0.050	48.000	1.315	0.195

SpCorr language (RH wholebrain) ~ within_between + (1|participant)

Spcorr within localizer vs. between localizer versions (right-hemisphere wholebrain), r^2 = 0.4235

	Estimate	Std. Error	df	t value	Pr(>|t|)
(Intercept)	0.206	0.060	53.012	3.406	0.001
Conditionwithin	0.015	0.056	48.000	0.265	0.792

Dice coefficient ~ within_between + (1|participant) + (1|fROI)

Dice within localizer vs. between localizer versions (top 10%), r^2 = 0.344

	Estimate	Std. Error	df	t value	Pr(>|t|)
(Intercept)	0.689	0.028	13.617	24.527	0.000
Conditionwithin	0.047	0.014	331.866	3.291	0.001

Dice within localizer vs. between localizer versions (top 20%), r^2 = 0.3726

	Estimate	Std. Error	df	t value	Pr(>|t|)
(Intercept)	0.750	0.023	13.771	32.806	0.000
Conditionwithin	0.025	0.011	331.882	2.281	0.023

Dice within localizer vs. between localizer versions (top 30%), r^2 = 0.361

	Estimate	Std. Error	df	t value	Pr(>|t|)
(Intercept)	0.785	0.019	15.668	40.276	0.000
Conditionwithin	0.012	0.010	331.870	1.238	0.217

Dice coefficient (LH wholebrain) ~ within_between + (1|participant) + (1|fROI)

Dice within localizer vs. between localizer versions (left-hemisphere wholebrain top 10%), r^2 = 0.0518

	Estimate	Std. Error	df	t value	Pr(>|t|)
(Intercept)	0.502	0.042	71.944	12.078	0.000
Conditionwithin	0.079	0.050	48.000	1.565	0.124

Dice within localizer vs. between localizer versions (left-hemisphere wholebrain top 20%), r^2 = 0.3757

	Estimate	Std. Error	df	t value	Pr(>|t|)
(Intercept)	0.953	0.020	56.158	46.970	0.000
Conditionwithin	-0.003	0.020	48.000	-0.130	0.897

Dice within localizer vs. between localizer versions (left-hemisphere wholebrain top 30%), r^2 = 0.2216

	Estimate	Std. Error	df	t value	Pr(>|t|)
(Intercept)	0.969	0.003	65.909	370.615	0.000
Conditionwithin	-0.002	0.003	48.000	-0.878	0.384

Dice coefficient (RH wholebrain) ~ within_between + (1|participant) + (1|fROI)

Dice within localizer vs. between localizer versions (right-hemisphere wholebrain top 10%), $r^2 = 0.1371$

	Estimate	Std. Error	df	t value	Pr(>|t|)
(Intercept)	0.513	0.036	69.680	14.337	0.000
Conditionwithin	0.036	0.041	48.000	0.875	0.386

Dice within localizer vs. between localizer versions (right-hemisphere wholebrain top 20%), $r^2 = 0.2916$

	Estimate	Std. Error	df	t value	Pr(>|t|)
(Intercept)	0.944	0.025	61.579	38.090	0.000
Conditionwithin	-0.008	0.026	48.000	-0.312	0.756

Dice within localizer vs. between localizer versions (right-hemisphere wholebrain top 30%), $r^2 = 0.0911$

	Estimate	Std. Error	df	t value	Pr(>|t|)
(Intercept)	0.966	0.003	70.960	335.496	0.000
Conditionwithin	-0.002	0.003	48.000	-0.688	0.495

SI 3: Information related to Results Section 3.1.2

SI 3A: Validation of hard > easy contrast from the MD localizer

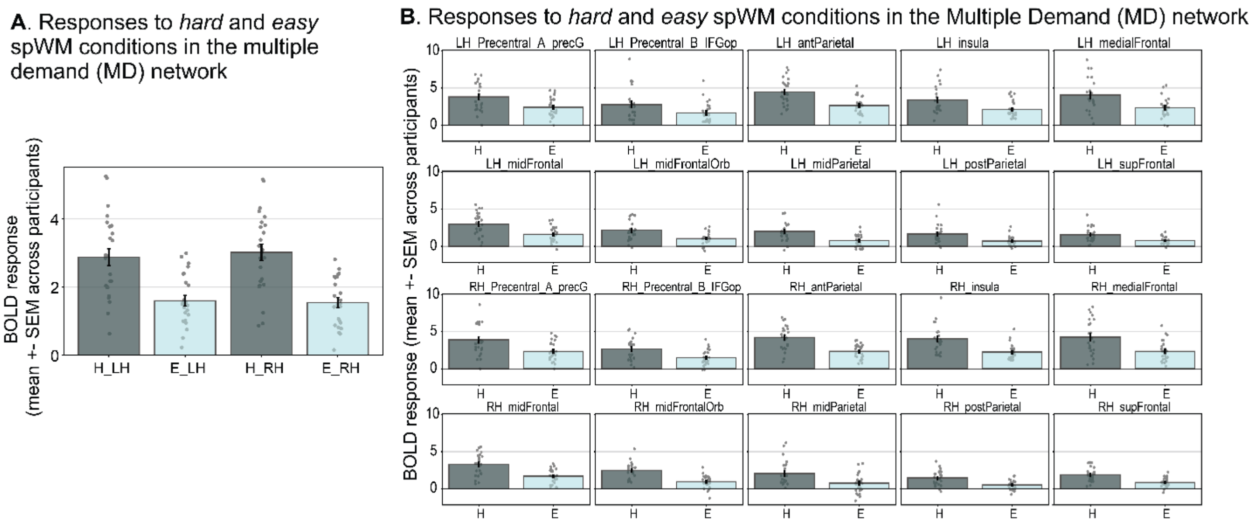

SI Figure 3A. Responses to the hard and easy spatial working memory (spWM) conditions in the MD localizer task.

(A) Mean response to the MD localizer conditions (H=*hard*, E=*easy*) averaged across ten Multiple Demand (MD) fROIs in each hemisphere. Dots show the mean response across fROIs of each individual participant. Error bars show the standard error of the mean across participants.
(B) Mean response to the MD localizer conditions for each MD fROI. Dots show the mean response in the particular fROI of each individual participant. Error bars show the standard error of the mean across participants.

SI 3B: Sentences > nonwords BOLD response magnitudes are highly correlated across runs for both the standard and speeded language localizer versions

To investigate how stable the *sentences* and *nonwords* BOLD responses were across individual scanning runs, we quantified the average BOLD response magnitudes of the *sentences* > *nonwords* contrast for each LH language fROI for the odd and even run of each localizer version separately.

Note that independent data were used to localize the fROI (i.e., data from the odd run were used to define the fROI, and responses were extracted from the even run, and vice versa).

The correlation between the average *sentence* > *nonwords* magnitude across LH language fROIs was greater in the standard language localizer than the speeded language localizer (**SI Figure 3B, panel A**). The correlation of the *sentences* > *nonwords* magnitude between odd and even runs across the five language fROIs was r = 0.82 (p<0.0001) for the standard language localizer, and r = 0.57 (p=0.0035) for the speeded language localizer. (Note that without the one outlier participant–bottom right in **SI Figure 3B, panel A**–the correlation was r = 0.83 for the speeded language localizer, p<0.0001).
The odd-even correlation values for individual fROIs were similarly high (**SI Figure 3B, panel B**): The average correlation across the five language fROIs was 0.823 (SD across fROIs: 0.062; five ps<0.001) for the standard language localizer, and 0.704 (SD: 0.083; five ps<0.001) for the speeded language localizer.

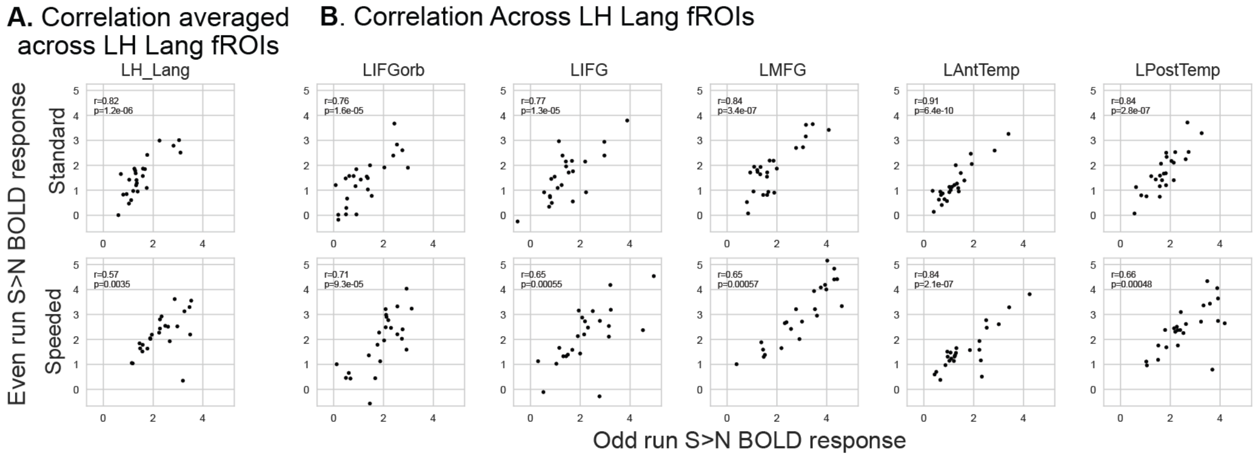

SI Figure 3B. Correlation of *sentences* > *nonwords* BOLD magnitudes within LH language fROIs obtained from odd and even runs.
(A) Correlation between *sentences* > *nonwords* BOLD magnitudes (averaged across the five LH language ROIs) of odd (x-axis) and even (y-axis) runs of the standard language localizer (upper row) and speeded language localizer (bottom row). Dots represent the *sentences* > *nonwords* BOLD response for each individual participant (*n*=24).
(B) Same as in panel A, just for each individual language fROI.

SI 3C: Consistency of localizers within participants across sessions

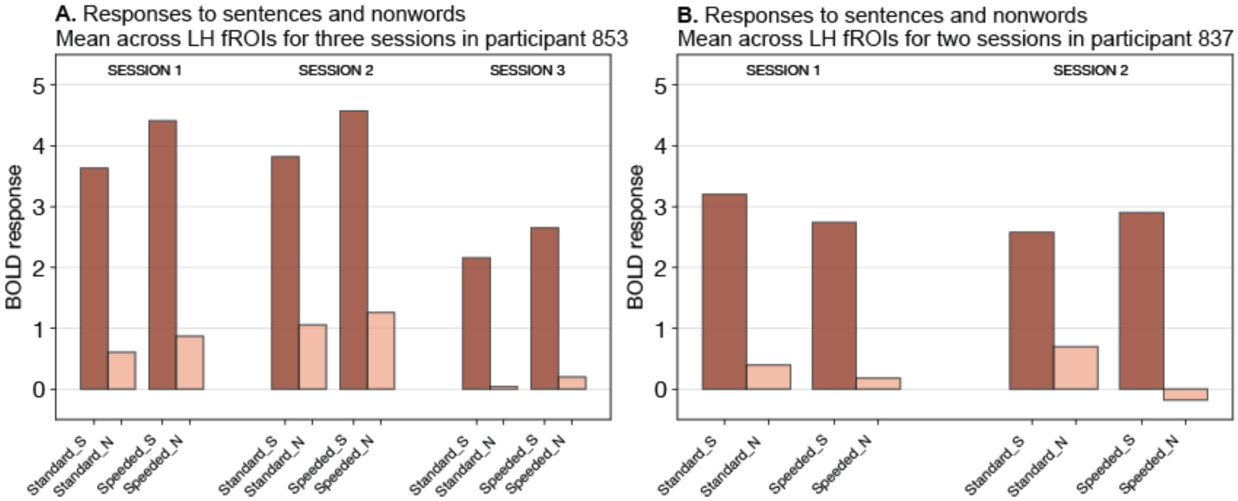

SI Figure 3C: Responses to the *sentences* and *nonwords* conditions in the standard and speeded language localizer for two participants across sessions.

Two participants in our dataset completed the two language localizers in different sessions (i.e., different days): One participant completed three sessions (the three sessions were 5 and 7 days apart, **panel A**), another participant completed two sessions (the two sessions were 99 days apart, **panel B**).

The mean responses to the language localizer conditions (S=*sentences*, N=*nonwords*) for each of the standard and speeded versions of the localizer task averaged across the five LH language fROIs are shown.

SI 3D: Statistics tables for Results Section 3.1.2 (responses to language)

In the tables below, "BOLD response" denotes the BOLD response magnitude for the given condition (*sentences, nonwords,* or *sentences* > *nonwords;* note that "language" denotes both *sentences* and *nonwords* responses). "condition" denotes the *sentences* and *nonwords* conditions in the LMEs where they are modeled together. "version" denotes the language localizer version, either standard or speeded. "participant" denotes each of the n=24 participants. "fROI" denotes each of the five LH fROIs.

(i) *BOLD response language ~ condition + version + (1|participant) + (1|fROI)*

Standard vs Speeded Without Interaction (LHLang), r^2 = 0.6515

	Estimate	Std. Error	df	t value	Pr(>|t|)
(Intercept)	0.442	0.270	7.587	1.636	0.142
ConditionS	1.897	0.080	451.954	23.570	0.000
VersionSpeeded	-0.068	0.080	451.954	-0.851	0.395

(ii) *BOLD response language ~ condition + version + condition:version + (1|participant) + (1|fROI)*

Standard vs Speeded With Interaction (LHLang), r^2 = 0.6671

	Estimate	Std. Error	df	t value	Pr(>|t|)
(Intercept)	0.623	0.273	7.895	2.282	0.052
ConditionS	1.535	0.111	451.955	13.801	0.000
VersionSpeeded	-0.430	0.111	451.955	-3.866	0.000
ConditionS:VersionSpeeded	0.723	0.157	451.955	4.597	0.000

(iii) *BOLD response sentences>nonwords ~ version + (1|participant) + (1|fROI)*

Standard vs Speeded S-N (LHLang), r^2 = 0.5214

	Estimate	Std. Error	df	t value	Pr(>|t|)
(Intercept)	1.535	0.204	10.980	7.521	0.000
VersionSpeeded	0.723	0.093	211.888	7.817	0.000

(iv) *BOLD response sentences ~ version + (1|participant) + (1|fROI)*

Standard vs Speeded S (LHLang), r^2 = 0.546

	Estimate	Std. Error	df	t value	Pr(>|t|)
(Intercept)	2.158	0.361	7.700	5.978	0.000
VersionSpeeded	0.293	0.122	211.940	2.407	0.017

(v) *BOLD response nonwords ~ version + (1|participant) + (1|fROI)*

Standard vs Speeded N (LHLang), r^2 = 0.3951

	Estimate	Std. Error	df	t value	Pr(>|t|)
(Intercept)	0.623	0.182	7.397	3.418	0.010
VersionSpeeded	-0.430	0.084	211.884	-5.106	0.000

SI 3E: Language BOLD responses for right hemisphere fROIs

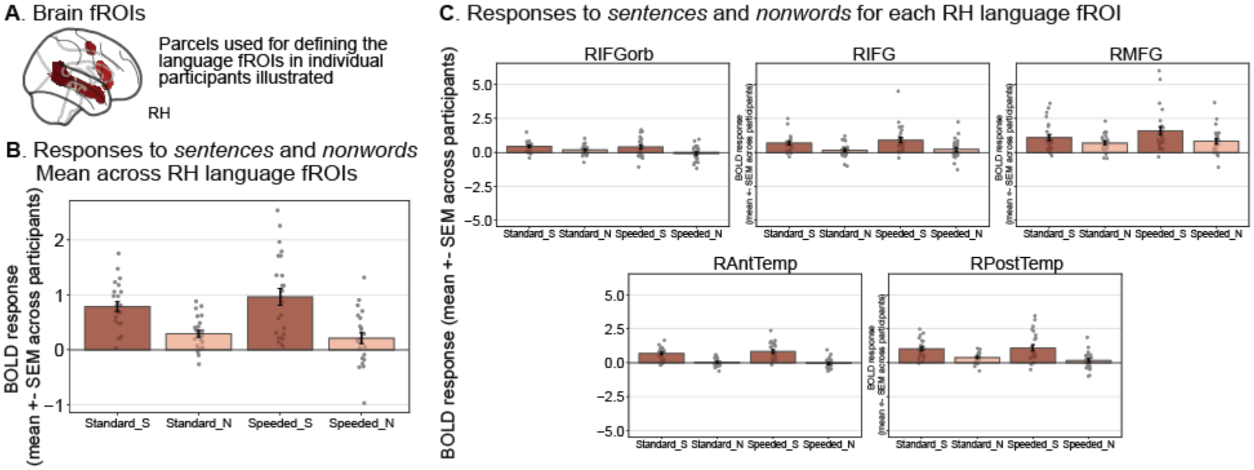

SI Figure 3E: Responses to the language localizer conditions (*sentences* and *nonwords*) for the standard and speeded language localizers in right hemisphere (RH) language fROIs.

(A) We defined the RH language fROIs as the most language-responsive voxels (top 10%) within the borders of the five anatomical parcels (see Methods; Extraction of fMRI BOLD responses) for each participant, and measured the BOLD response magnitude in these fROIs in a cross-validated manner (see Methods; Definition of fROIs).

(B) Mean BOLD response to the language localizer conditions (S=*sentences*, N=*nonwords*) for both the standard and speeded localizer versions averaged across the five RH language fROIs.
(C) Mean BOLD response to the localizer conditions for each RH language fROI. In both panels, dots show the mean response of each individual participant. Error bars show the standard error of the mean across participants.

The statistics tables accompanying SI Figure 3E are found below.

(i) *BOLD response RH language ~ condition + version + (1|participant) + (1|fROI)*

Standard vs Speeded Without Interaction (RHLang), $r^2 = 0.32751$

	Estimate	Std. Error	df	t value	Pr(>|t|)
(Intercept)	0.228	0.146	8.359	1.555	0.157
ConditionS	0.623	0.065	451.888	9.617	0.000
VersionSpeeded	0.047	0.065	451.888	0.732	0.465

(ii) *BOLD response RH language ~ condition + version + condition:version + (1|participant) + (1|fROI)*

Standard vs Speeded With Interaction (RHLang), $r^2 = 0.3332$

	Estimate	Std. Error	df	t value	Pr(>|t|)
(Intercept)	0.291	0.150	9.180	1.942	0.083
ConditionS	0.496	0.091	451.889	5.438	0.000
VersionSpeeded	-0.079	0.091	451.889	-0.872	0.384
ConditionS:VersionSpeeded	0.254	0.129	451.889	1.968	0.050

(iii) *BOLD response RH sentences>nonwords delta ~ version + (1|participant) + (1|fROI)*

Standard vs Speeded S-N (RHLang), $r^2 = 0.3587$

	Estimate	Std. Error	df	t value	Pr(>|t|)
(Intercept)	0.496	0.105	17.126	4.717	0.000
VersionSpeeded	0.254	0.067	211.758	3.776	0.000

(iv) *BOLD response RH sentences ~ version + (1|participant) + (1|fROI)*

Standard vs Speeded S (RHLang), $r^2 = 0.2493$

	Estimate	Std. Error	df	t value	Pr(>|t|)
(Intercept)	0.787	0.168	9.317	4.678	0.001
VersionSpeeded	0.174	0.106	211.768	1.651	0.100

(v) *BOLD response RH nonwords ~ version + (1|participant) + (1|fROI)*

Standard vs Speeded N (RHLang), $r^2 = 0.2648$

	Estimate	Std. Error	df	t value	Pr(>|t|)
(Intercept)	0.291	0.134	7.002	2.175	0.066
VersionSpeeded	-0.079	0.071	211.842	-1.118	0.265

SI 3F: Statistics tables for Results Section 3.1.2 (responses to working memory task)

In the tables below, "BOLD response" denotes the BOLD response magnitude for the given condition (*hard, easy*). "version" denotes the language localizer version, either standard or

speeded. "participant" denotes each of the n=24 participants. "fROI" denotes each of the five LH fROIs.

i) *BOLD response hard ~ version + (1|participant) + (1|fROI)*

Standard vs Speeded H (LHLang), $r^2 = 0.549$

	Estimate	Std. Error	df	t value	Pr(>|t|)
(Intercept)	-0.383	0.284	9.648	-1.347	0.209
VersionSpeeded	0.101	0.104	211.929	0.973	0.332

ii) *BOLD response easy ~ version + (1|participant) + (1|fROI)*

Standard vs Speeded E (LHLang), $r^2 = 0.4984$

	Estimate	Std. Error	df	t value	Pr(>|t|)
(Intercept)	-0.054	0.156	13.538	-0.343	0.737
VersionSpeeded	0.096	0.070	211.891	1.361	0.175

SI 3G: Language responses in extended language network fROIs

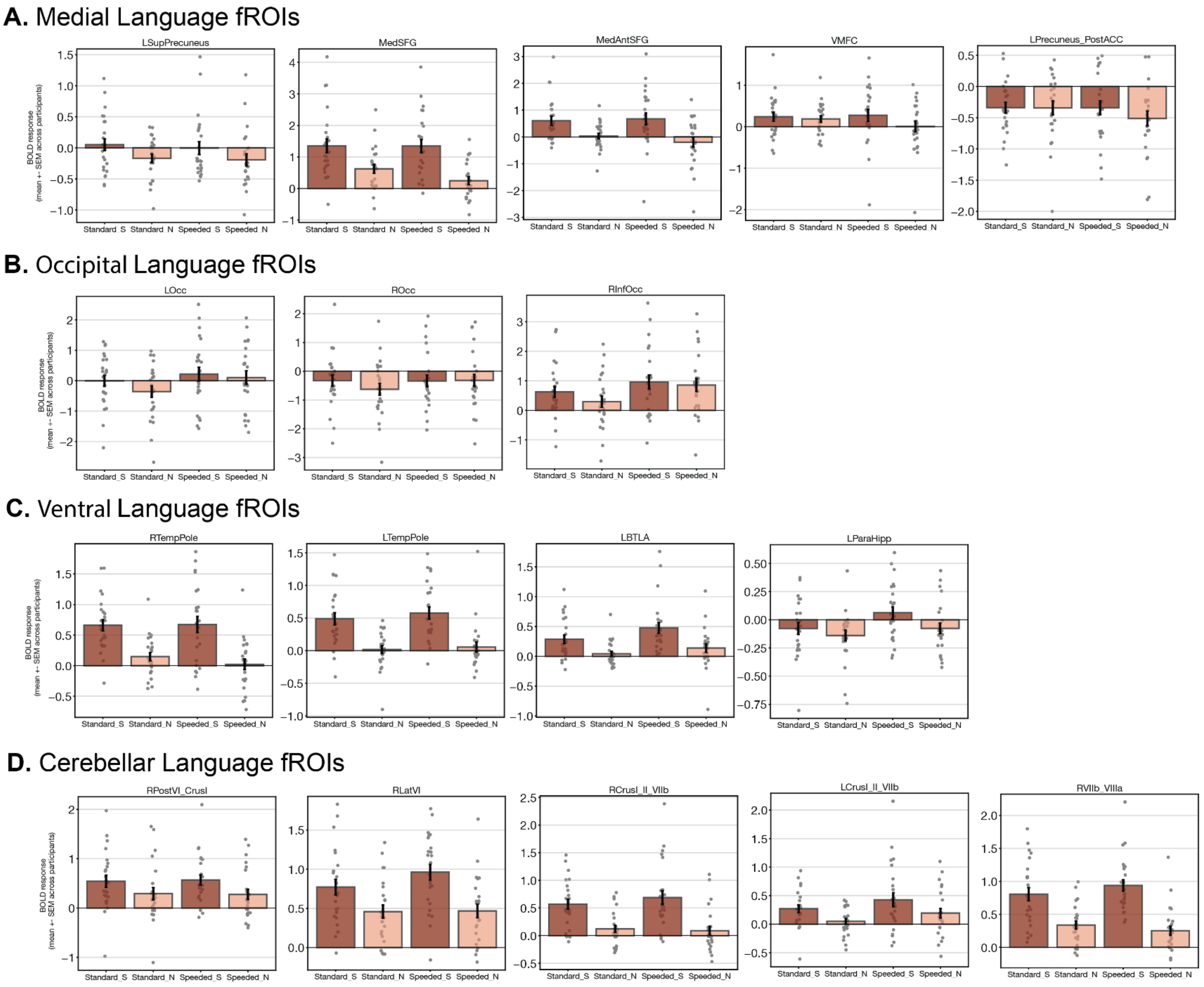

SI Figure 3G. **Responses to the standard and speeded localizer tasks in the extended language network.** Mean BOLD response to the language localizer conditions (S=sentences, N=nonwords) for each of the standard and speeded versions of the localizer task for each fROI in the **(A)** Medial, **(B)** Occipital, **(C)** Ventral, and **(D)** Cerebellar regions of the extended language network as described by Wolna et al. (2025). Dots show the mean response across fROIs of each individual participant. Error bars show the standard error of the mean across participants.

SI 3H: Language responses in DMN fROIs

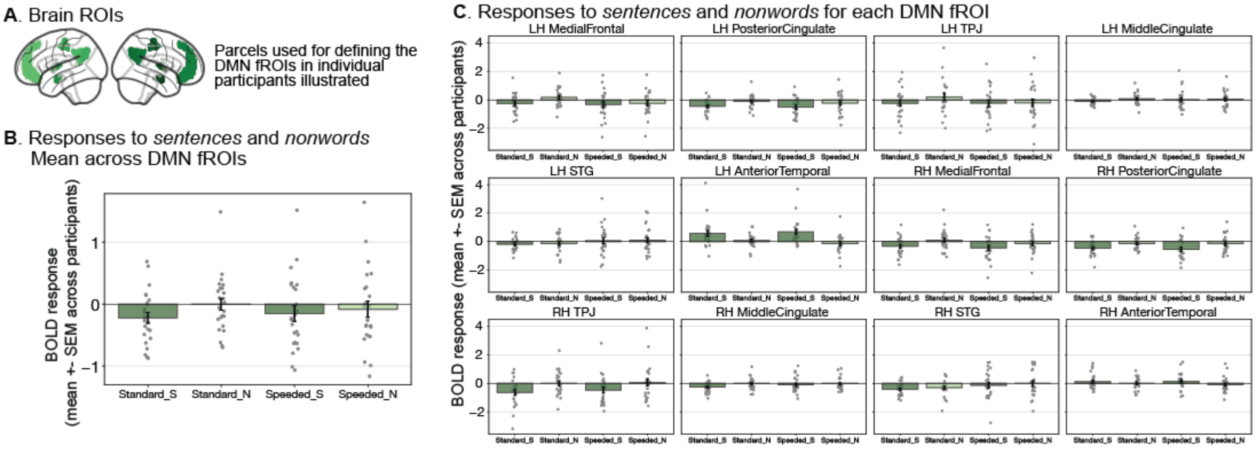

(A) To define the DMN fROIs, we used a set of 12 parcels (6 in each hemisphere) derived from a group-level probabilistic activation overlap map for the *easy* > *hard* spatial working memory contrast (Fedorenko et al., 2013) in 197 independent participants. The *easy* > *hard* contrast has been shown to robustly activate DMN regions, in line with prior work using similar tasks and contrasts (Leech et al., 2011; Mineroff, Blank et al., 2018). The parcels included symmetrical regions in the posterior cingulate cortex, four medial frontal regions, the precuneus, and temporoparietal junction. We used the *easy* > *hard* spatial working memory (WM) contrast to identify the top 10% most responsive voxels within these parcels, and extracted responses to sentences and nonwords conditions within these fROIs for both the standard and speeded language localizer versions. We measured the BOLD response magnitude to the language localizer conditions in these fROIs in a cross-validated manner (see Methods; Definition of fROIs).

(B) Mean BOLD response to the language localizer conditions (S=sentences, N=nonwords) for both the standard and speeded localizer versions averaged across the DMN fROIs. On average, the DMN parcels demonstrated a *nonwords* > *sentences* effect (*sentences* > *nonwords*, β=-0.218, t=-3.913, p<0.0001).

(C) Mean BOLD response to the localizer conditions for each DMN fROIs. In both panels, dots show the mean response of each individual participant. Error bars show the standard error of the mean across participants. With the exception of the left anterior-temporal region, all DMN regions demonstrated a *nonwords* > *sentences* response. Notably, this left anterior-temporal region lies within the left anterior-temporal region of the language parcel, and hence the positive *sentences* > *nonwords* response is likely explained by language-selective voxels in this region.

BOLD response standard language ~ condition + (1|participant) + (1|fROI)

S-N Standard (DMN), R-Squared = 0.4035

	Estimate	Std. Error	df	t value	Pr(>|t|)
(Intercept)	-0.005	0.100	34.967	-0.047	0.963
ConditionS	-0.218	0.045	540.891	-4.858	0.000

BOLD response speeded language ~ condition + (1|participant) + (1|fROI)

S-N Speeded (DMN), R-Squared = 0.4904

	Estimate	Std. Error	df	t value	Pr(>|t|)
(Intercept)	-0.084	0.134	32.106	-0.624	0.537
ConditionS	-0.071	0.052	540.921	-1.372	0.170

BOLD response language ~ condition + version + condition:version + (1|participant) + (1|fROI)

Standard vs. Speeded With Interaction (DMN), R-Squared = 0.2778

	Estimate	Std. Error	df	t value	Pr(>|t|)
(Intercept)	-0.005	0.098	41.296	-0.048	0.962
ConditionS	-0.218	0.056	1116.905	-3.913	0.000
VersionSpeeded	-0.079	0.056	1116.905	-1.415	0.157
ConditionS:VersionSpeeded	0.147	0.079	1116.905	1.867	0.062

SI 4: Information related to Results Section 3.2 (Multiple Demand network)

SI 4A: MD responses to the language localizer versions across fROIs

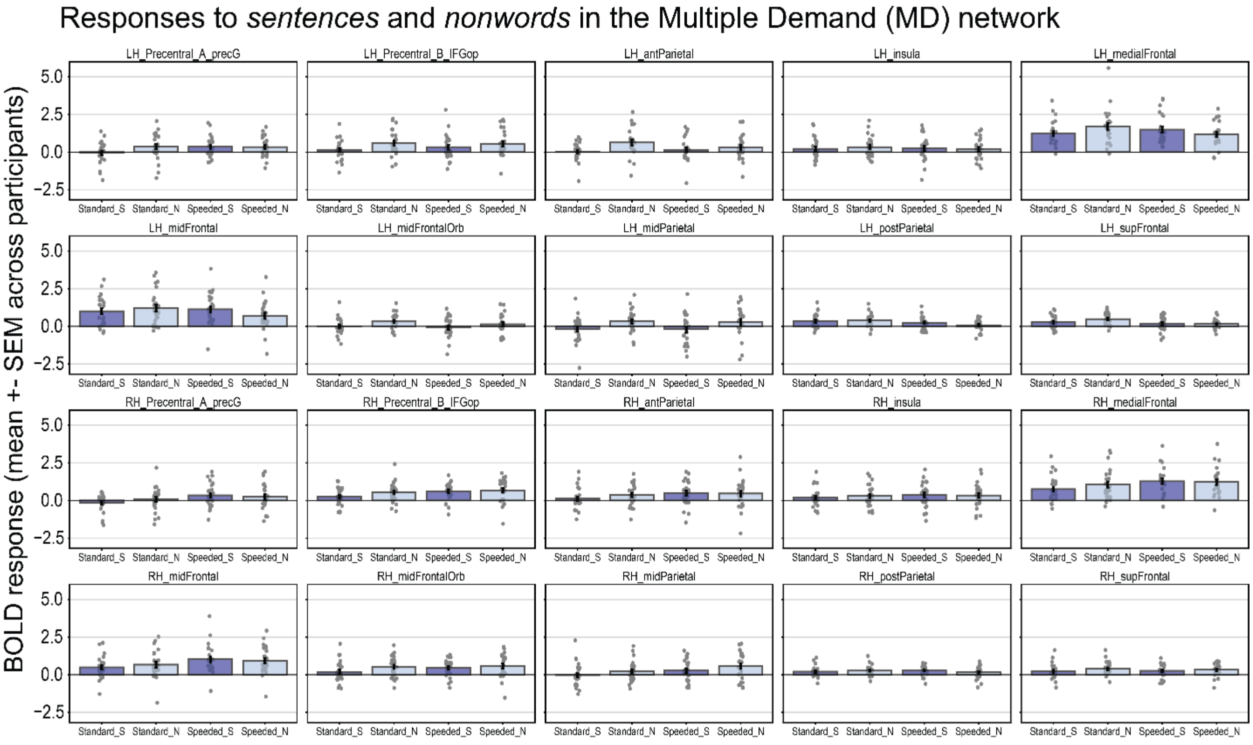

SI Figure 4A. Responses to the standard and speeded localizer tasks in the Multiple Demand Region across all twenty left and right hemispheric MD fROIs (ten in each hemisphere). Mean BOLD response to the language localizer conditions (S=*sentences,* N=*nonwords*) for each of the standard and speeded versions of the localizer task for each Multiple Demand (MD) fROI in the left and right hemispheres. Dots

show the mean response across fROIs of each individual participant. Error bars show the standard error of the mean across participants.

SI 4B: Statistics tables for Results Section 3.2

In the tables below, "BOLD response" denotes the BOLD response magnitude for the given condition (*sentences, nonwords*; note that "language" denotes both *sentences* and *nonwords* responses). "condition" denotes the *sentences* and *nonwords* conditions in the LMEs where they are modeled together. "version" denotes the language localizer version, either standard or speeded. "participant" denotes each of the *n*=24 participants. "fROI" denotes each of the twenty LH/RH MD fROIs (ten in each hemisphere).

BOLD response standard language ~ condition + (1|participant) + (1|fROI)

S-N Standard (MD), $r^2 = 0.5257$

	Estimate	Std. Error	df	t value	Pr(>|t|)
(Intercept)	0.541	0.125	42.783	4.325	0.000
ConditionS	-0.275	0.036	916.956	-7.547	0.000

BOLD response speeded language ~ condition + (1|participant) + (1|fROI)

S-N Speeded (MD), $r^2 = 0.44$

	Estimate	Std. Error	df	t value	Pr(>|t|)
(Intercept)	0.477	0.123	42.998	3.864	0.000
ConditionS	-0.012	0.041	916.942	-0.283	0.777

BOLD response language ~ condition + version + condition:version + (1|participant) + (1|fROI)

Standard vs. Speeded With Interaction (MD), $r^2 = 0.3504$

	Estimate	Std. Error	df	t value	Pr(>|t|)
(Intercept)	0.541	0.108	45.171	5.002	0.000
ConditionS	-0.275	0.044	1876.956	-6.328	0.000
VersionSpeeded	-0.064	0.044	1876.956	-1.470	0.142
ConditionS:VersionSpeeded	0.264	0.062	1876.956	4.285	0.000

BOLD response sentences ~ version + (1|participant) + (1|fROI)

Standard vs. Speeded S (MD), $r^2 = 0.3263$

	Estimate	Std. Error	df	t value	Pr(>|t|)
(Intercept)	0.266	0.104	36.377	2.549	0.015
VersionSpeeded	0.200	0.044	916.907	4.584	0.000

BOLD response nonwords ~ version + (1|participant) + (1|fROI)

Standard vs. Speeded N (MD), $r^2 = 0.4024$

	Estimate	Std. Error	df	t value	Pr(>|t|)
(Intercept)	0.541	0.117	42.660	4.634	0.000
VersionSpeeded	-0.064	0.042	916.932	-1.519	0.129

Supplemental Information References

Fedorenko, E., Duncan, J., & Kanwisher, N. (2013). Broad domain generality in focal regions of frontal and parietal cortex. *Proceedings of the National Academy of Sciences*, *110*(41), 16616–16621. https://doi.org/10.1073/pnas.1315235110

Leech, R., Kamourieh, S., Beckmann, C. F., & Sharp, D. J. (2011). Fractionating the default mode network: distinct contributions of the ventral and dorsal posterior cingulate cortex to cognitive control. *Journal of Neuroscience*, 31(9), 3217-3224.

Mineroff, Z., Blank, I. A., Mahowald, K., & Fedorenko, E. (2018). A robust dissociation among the language, multiple demand, and default mode networks: Evidence from inter-region correlations in effect size. *Neuropsychologia*, *119*, 501–511. https://doi.org/10.1016/j.neuropsychologia.2018.09.011

Wolna, A., Wright, A., Casto, C., Lipkin, B., & Fedorenko, E. (2025). The extended language network: Language selective brain areas whose contributions to language remain to be discovered. *bioRxiv*, doi: https://doi.org/10.1101/2025.04.02.646835
